# EAR-20 peptide, a novel NMDA receptor positive allosteric modulator

**DOI:** 10.3389/fphar.2025.1639698

**Published:** 2025-11-21

**Authors:** Roberto García-Díaz, Aida Castellanos, Federico Miguez-Cabello, Javier Picañol, Anna Priscil·la Pérez-González, Esther Gratacòs-Batlle, Nohora Vega-Castro, Xavier Altafaj, Edwin A. Reyes-Guzmán, Edgar A. Reyes-Montaño, Xavier Gasull, David Soto

**Affiliations:** 1 Neurophysiology Laboratory, Department of Biomedicine, Faculty of Medicine and Health Sciences, Institute of Neurosciences, University of Barcelona, Barcelona, Spain; 2 Grupo de Investigación en Proteínas, Departamento de Química, Universidad Nacional de Colombia, Bogotá, Colombia; 3 Facultad de Ingeniería, Programa de Ingeniería Biomédica, Universidad Militar Nueva Granada, Bogotá, Colombia; 4 Fundamental and Clinical Nursing Department, Faculty of Nursing, Institute of Neurosciences, University of Barcelona, Barcelona, Spain; 5 August Pi i Sunyer Biomedical Research Institute (IDIBAPS), Barcelona, Spain; 6 Doctorado en Ciencia Aplicada, Universidad Antonio Nariño, Bogotá, Colombia

**Keywords:** NMDARs, positive allosteric modulator, whole-cell patch-clamp, hippocampal neurons, single-channel, molecular docking

## Abstract

Allosteric modulation of ligand-gated ion channels provides a powerful mechanism to fine-tune their activity without competing with endogenous ligands. In the case of NMDA receptors (NMDARs), which are critical for excitatory neurotransmission and synaptic plasticity, allosteric modulators represent potential therapeutic tools, particularly in conditions involving NMDAR hypofunction. Here, we characterize EAR-20, a 17-amino-acid peptide derived from the marine cone snail toxin Conantokin-G, as a novel positive allosteric modulator (PAM) of NMDARs. Using molecular docking, whole-cell and single-channel patch-clamp electrophysiology, and recordings in cultured hippocampal neurons, we show that EAR-20 enhances receptor function by increasing channel open probability and reducing desensitization, and can even activate NMDARs in the absence of exogenous glutamate and glycine, albeit to a lower extent. EAR-20 decreased desensitization, potentiating GluN1-GluN2A and GluN1-GluN2B receptors more than twofold, modestly enhanced (∼25%) GluN1-GluN2A-GluN2B tri-heteromers, and increased NMDAR-mediated currents in primary hippocampal neurons. Molecular docking identified a binding site at the GluN1-GluN2B interface, with Ser773 in GluN1 being critical for the modulatory effect. Importantly, EAR-20 partially rescued hypofunctional NMDARs carrying patient-derived loss-of-function mutations. Together, these findings identify EAR-20 as a novel subunit-dependent positive allosteric modulator with the potential to inspire the development of small molecules targeting the same binding site, offering proof of concept for therapeutic strategies to treat neurological and neurodevelopmental disorders.

## Introduction

The amino acid glutamate is the main excitatory neurotransmitter in the mammalian central nervous system (CNS) (Zhou and Danbolt, 2014) acting on two types of receptors: ionotropic receptors (cation-permeable ligand-gated ion channels; iGluRs) and metabotropic receptors (G protein-coupled receptors; mGluRs) (Reiner and Levitz, 2018; Hansen et al., 2021). While metabotropic receptors mediate long-term modulatory effects (Gregory et al., 2013), iGluRs are responsible for the fast glutamatergic neurotransmission in the CNS, which is essential for normal brain development and function (Hansen et al., 2021; Collingridge and Abraham, 2022). iGluRs can be classified into three subfamilies based on gene sequence similarities: AMPA receptors (composed of GluA1–4 subunits), kainate receptors (composed of GluK1–5 subunits), and NMDA receptors (composed of GluN1, GluN2A–D, and GluN3A–B subunits), each with distinct biophysical properties and functional roles in the CNS ((Hansen et al., 2021).

NMDARs are heterotetramers composed of four subunits that can be assembled into either di-heteromeric or tri-heteromeric forms with two obligatory glycine-binding GluN1 subunits and two other identical or different glutamate-binding GluN2 subunits, namely GluN2A, GluN2B, GluN2C, or GluN2D (Salussolia et al., 2011; Hansen et al., 2021). Each NMDAR subunit is composed of four distinct domains: the amino-terminal domain (ATD), the ligand-binding domain (LBD), the transmembrane domain (TMD), and the carboxy-terminal domain (CTD) (Karakas and Furukawa, 2014). Subunit composition provides unique characteristics to each type of NMDAR, resulting in different functional properties including the open time of the channel, channel conductance, permeability to Ca^2+^, sensitivity to blockage by Mg^2+^, affinity for agonists glycine or D-serine and L-glutamate, and sensitivity and sensibility to allosteric modulators (Wyllie et al., 2013; Zhou and Tajima, 2023).

NMDA receptors are unique among glutamate-gated ion channels because they require the binding of two agonists–glutamate and glycine (or D-serine) – in addition to membrane depolarization for activation. At resting membrane potentials, extracellular Mg^2+^ ions enter and occupy a site in the channel pore, producing a strong voltage-dependent block. When synaptic depolarization occurs (typically through AMPAR activation), the driving force for Mg^2+^ entry is reduced and the ion is expelled from the pore, thereby relieving the block and allowing current to flow (Nowak et al., 1984; Jahr and Stevens, 1990). Besides, NMDARs also differ from other ionotropic glutamate receptors in their higher affinity for glutamate, a property that contributes to their relatively slow activation kinetics (Hansen et al., 2017). On the other side, the glycine-binding site, located on the GluN1 subunit, has an even higher affinity and very low concentrations of glycine are enough to saturate it (Johnson and Ascher, 1987). In fact, ambient extracellular glycine is often sufficient for activating them, although at many forebrain synapses D-serine is the predominant co-agonist (Papouin et al., 2012). Taken together, these properties establish NMDA receptors as coincidence detectors that respond only when presynaptic glutamate release coincides with postsynaptic depolarization (Seeburg et al., 1995).

NMDARs not only contribute to fast excitatory synaptic transmission but also trigger signaling events upon Ca^2+^ entry through their channel pore. These calcium-mediated processes are essential for the activation of physiological functions such as neuronal development and synaptic plasticity, which are fundamental to learning and memory processes (Hansen et al., 2021; Collingridge and Abraham, 2022). Their crucial role is highlighted in numerous studies showing that dysfunction of NMDARs–due to hypo- or hyperactivation–is associated with neurodevelopmental disorders and neurological diseases (Gandal et al., 2012; Volk et al., 2015; Yamamoto et al., 2015; Tumdam et al., 2024). One example of such importance is found in a group of neurodevelopmental disorders known as GRIN-related disorders, resulting from mutations of GRIN genes that encode for the GluN2 subunits of the NMDAR. These genetic alterations lead to intellectual disability along with other neurological and neurodevelopmental impairments (Endele et al., 2010; Benke et al., 2021). The pathological outcome of primary and secondary NMDAR dysfunction stimulated pharmacological research over the years, testing the use of agonists and antagonists, such as memantine, for treating certain NMDAR related disorders (Zhang et al., 2024). Although memantine’s side effects are generally mild (Parsons et al., 1999), agonists (e.g. glutamate analogs) and antagonists (e.g. ketamine or PCP) of NMDARs can cause more serious side effects, such as dissociative symptoms, cognitive impairment, psychosis, seizures or even cardiovascular problems (Kaindl and Ikonomidou, 2007; Short et al., 2018; Moore et al., 2022). That turned special attention on NMDAR allosteric modulators, both positive (PAMs) and negative (NAMs), as a safer and more effective alternative as a potential therapeutic strategy for these neurological disorders (Menniti et al., 2013; Geoffroy et al., 2022). Interestingly, PAMs ability to enhance NMDAR activity might be suitable for personalized medicine in patients harboring GRIN loss-of-function, potentially restoring or mitigating these deleterious effects (Tang et al., 2020; Santos-Gómez et al., 2025).

The EAR-20 peptide was originally designed as a scrambled control peptide derived from a set of modified peptides based on Conantokin-G (Con-G), a well-known NMDAR antagonist, which specifically targets the GluN2B ligand binding domain. These modified peptides were developed as part of a strategy to finely regulate NMDAR activity through structural variants inspired by Con-G. EAR-20 is not a direct scrambled version of Con-G itself, but rather a scrambled sequence of one of the Con-G–based modified peptides (*see methods*), and initially, the EAR-20 peptide was intended as a control for electrophysiological tests. Surprisingly, it was observed that EAR-20 activated currents in hippocampal neurons in the absence of added glycine and glutamate (Reyes-Guzman et al., 2017). This unexpected finding prompted us to further investigate the effects of EAR-20 on NMDA receptors using electrophysiological techniques. In recombinant receptors expressed in HEK-293T cell line, we have found that EAR-20 peptide exhibits a small but significant agonistic activity in GluN1-GluN2B di-heteromers, as well as a considerable PAM activity of GluN1-GluN2A, GluN1-GluN2B and GluN1-GluN2D receptors. This allosteric interaction enhances the effects of the co-agonists glutamate and glycine by increasing the channel open probability. EAR-20 peptide also potentiates NMDARs in mouse hippocampal neurons, suggesting that the peptide may act as a functional PAM in native receptors. Moreover, EAR-20 exerts a rescuing effect on hypofunctional NMDARs, as tested in loss-of-function variants where receptor activity is compromised, further supporting its role as a functional modulator with potential therapeutic relevance.

## Methods

### Peptide synthesis

EAR-20 peptide (^1^KLGMRSELQIDNDQDAD^17^) was synthesized manually by solid-phase peptide synthesis (SPPS), using for this purpose the alpha nitrogen of the amino acids protected with the base labile Fmoc group (Behrendt et al., 2016) as described in García Díaz (2024). The synthesis method is also detailed in Reyes-Montaño et al. (2023).

### Plasmids

The expression plasmids for rat GluN1 and GFP-GluN2A and GFP-GluN2B were provided by S. Vicini (Georgetown University Medical Center, Washington, United States). Dr. J.W. Johnson (University of Pittsburgh, Pittsburgh, United States of America) provided HA-GluN2C and HA-GluN2D plasmids. The plasmids used to analyze triheteromeric NMDARs and N-terminal-deleted GluN2 (GluN2^ΔNTD^) were kindly provided by Dr. Pierre Paoletti [École Normale Supérieure Paris, France, EU (Stroebel et al., 2014; Rachline et al., 2005)]. Nucleotide changes for producing GRIN variants were achieved by oligonucleotide-directed mutagenesis, using the QuikChange II XL protocol with turbo Pfu DNA polymerase (Stratagene La Jolla, CA, United States) to replicate the parental DNA strand with the desired mismatch incorporated into the primer. Methylated parental DNA was digested with DpnI for 1 h at 37 °C, and the nicked mutant DNA was transformed into XL1-Blue Super Competent Cells (Stratagene La Jolla, CA, United States). The Bacteria were spun down, and plasmid DNA isolated using the Qiagen Spin Miniprep kit (Hilden, Germany). Sequences were verified by Sanger sequencing (STAB vida, Caparica, Portugal).

### Cell culture and transfection

HEK-293T cell lines were obtained from the American Type Culture Collection and maintained in Dulbecco’s modified Eagle’s medium (DMEM), supplemented with 10% fetal bovine serum and 1% penicillin (100 U/mL) and streptomycin (100 μg/mL) at 37 °C and 5% CO_2_. HEK-293T cells were transfected with a total of 1.3 μg of cDNA constructs that encode for wild-type GluN1 and GluN2 subunits or GluN1 and GluN2^ΔNTD^ subunits as well as green fluorescent protein (GFP) (at a ratio of 1:2:0.25 for GluN1: GluN2: GFP) mixed with 1 μL Lipofectamine 2000 reagent (Invitrogen) following the manufacturer’s instructions. Following transfection, NMDAR antagonists D-AP5 (D-2-amino-5-phosphonopentanoic acid, 500 μM) and 7-CKA (5,7-dichlorokynurenic acid, 200 μM) were added to the culture medium to reduce cell death caused by excessive NMDAR activation. The cells were used for electrophysiological recordings 24–48 h after transfection.

### Hippocampal cultures

Pregnant rats were kept and used in accordance with protocols approved by the Ethics Committee of the University of Barcelona. Pregnant rats were killed using CO_2_ followed by cervical dislocation prepared as previously described (Soto et al., 2019). Day 18 embryos (E18) were isolated and maintained in cold Hank’s Balanced Salt Solution (HBSS, Gibco) supplemented with 0.45% glucose (HBSS-Glucose) until dissection. Embryos were decapitated, skull was opened and meninges were carefully removed. The hippocampi were then collected and mildly digested with trypsin, washed in HBSS and resuspended in Neurobasal medium supplemented with 2 mM Glutamax (Gibco). Then, the cells were filtered through 70 mm mesh filters (BD Falcon). Isolated cells were then plated onto glass coverslips (5 × 10^4^ cells/cm^2^) coated with 0.1 mg/mL poly-L-lysine (Sigma) and 2 h after seeding, the plating medium was substituted by complete growth medium (Neurobasal medium supplemented with 2% B27 and 2 mM Glutamax). Primary cultures were incubated at 37 °C in a humidified atmosphere of 5% CO_2_. Every 3–4 days, half of the medium was removed and replaced by fresh growth medium. Cultures were used for patch clamp experiments between DIV17 and DIV19.

### Molecular docking simulation

Molecular docking simulations were performed using AutoDock Vina (Trott and Olson, 2010) to predict the interaction between the EAR-20 peptide and the rat NMDA receptor (NMDAR). The X-ray crystal structure of the full GluN1a-GluN2B receptor (4PE5; Karakas and Furukawa, 2014) was used as the receptor model. This structure was prepared using PyMOL (The PyMOL Molecular Graphics System, Schrödinger LLC. Original Invoice Number Registered: 57029) by removing water molecules, ions, and cofactors. Hydrogen atoms were then added and Kollman charges were assigned using AutoDock Tools. The three-dimensional structure of the EAR-20 peptide (17 amino acids), for which there is no experimental structural data, was predicted using three modeling tools: PEP-FOLD4, I-TASSER, and pepstrMOD. Based on the discrete optimized protein energy score (DOPE score), the PEP-FOLD4 structure was selected. The PEP-FOLD4 model was inspected by visualization in PyMOL and the sidechain stereochemistry was evaluated by Ramachandran plots (Ramachandran et al., 1963) on the PROSA web server (Wiederstein and Sippl, 2007).

Docking was directed to the GluN1/GluN2B ligand-binding domain (LBD), based on blind docking predictions using CD-DOCK and functional data indicating that the ATD was not involved in EAR-20 activity. With the help of algorithm AUTOGRID we were able to centralize the grid boxes at (0 Å, 60 Å, −20 Å) with sizes of 36 Å in each dimension, with a grid spacing of 0.4 Å, while keeping the NMDAR rigid and the EAR-20 peptide flexible. To validate the docking parameters, we performed control docking with glutamate on the same receptor model, yielding interactions consistent with known binding residues. The interaction energy between the EAR-20 peptide and the NMDAR was calculated for the entire binding site and expressed as affinity (kcal/mol). The binding interactions between the protein and ligands were further visualized and analyzed using PyMOL. Serine 773 and Serine 189 from GluN1, as well as Aspartate 524 from GluN2B, were identified as crucial residues for ligand binding and were selected for mutagenesis.

### Electrophysiological recordings of NMDAR whole-cell currents in HEK293T cells

The macroscopic response time course of NMDAR responses was determined from whole-cell patch recording of transiently transfected HEK cells or primary cultured hippocampal neurons. Electrophysiological recordings were obtained 24–48 h after transfection, perfusing the cells continuously at room temperature with extracellular physiological bath solution (in mM): 140 NaCl, 5 KCl, 1 CaCl_2_, 10 glucose, and 10 HEPES, adjusted to pH 7.40 with NaOH. Glutamate (1 mM, Sigma-Aldrich), in the presence of glycine (50 μM; Tocris) was applied for 5 s by piezoelectric translation (P-601.30; Physik Instrumente) of a theta-barrel application tool made from borosilicate glass (1.5 mm o.d.; Sutter Instruments). The activated currents were recorded in the whole-cell configuration at a holding potential of −60 mV, acquired at 5 kHz and filtered at 2 kHz by means of Axopatch 200B amplifier, Digidata 1440A interface and pClamp10 software (Molecular Devices Corporation). Electrodes with open-tip resistances of 2–4 MΩ were made from borosilicate glass (1.5 mm o.d., 0.86 mm i.d., Harvard Apparatus), pulled with a PC-10 vertical puller (Narishige) and filled with intracellular pipette solution containing (in mM): 140 CsCl, 5 EGTA, 4 Na_2_ATP, 0.1 Na_3_GTP and 10 HEPES, adjusted to pH 7.25 with CsOH. Glutamate and glycine-evoked currents were expressed in pA. The kinetics of desensitization of the NMDAR responses were determined by fitting the glutamate/glycine-evoked responses at *V*_*m*_ −60 mV to a double-exponential function in order to determine the weighted time constant (τ_*w*,des_):

$$\tau_{w,des}=\tau_{f}\left(\frac{A_{f}}{A_{f}+A_{s}}\right)+\tau_{s}\left(\frac{A_{s}}{A_{f}+A_{s}}\right)$$

where A_f_ and τ_f_ are the amplitude and time constant of the fast component of desensitization and A_s_ and τ_s_ are the amplitude and time constant of the slow component of desensitization. Quantitative analysis of the effects of the EAR-20 peptide was performed by comparing the peak amplitude and steady plateau elicited by the application of 1 mM L-glutamate plus 50 μM glycine, supplemented with 100 μM EAR-20, and normalized to the response in the presence of glutamate plus glycine condition.

### Electrophysiological recordings of hippocampal neurons

Whole-cell voltage-clamp recordings were performed on cultured hippocampal neurons at room temperature using an Axopatch 200B amplifier (Molecular Devices). Patch pipettes (resistance 3–5 MΩ) were pulled from borosilicate glass and filled with an internal solution containing (in mM): 140 CsCl, 5 EGTA, 4 Na_2_ATP, 0.1 Na_3_GTP and 10 HEPES, adjusted to pH 7.25 with CsOH. QX-314 (250 µM) was included in the pipette solution to block potential firing. The external recording solution contained (in mM): 140 NaCl, 5 KCl, 1 CaCl_2_, 10 glucose, and 10 HEPES, adjusted to pH 7.40 with NaOH. To isolate the NMDAR component, 10 μM NBQX and 50 μM picrotoxin were added to the external recording solution to block AMPAR and GABAR-mediated EPSCs, respectively. Neurons were held at a membrane potential of −60 mV, and NMDAR-mediated currents were evoked by brief local application of 100 µM glutamate using a fast perfusion system. Evoked responses were recorded in both control and EAR-20 conditions at a final concentration of 100 μM, and currents were compared before and during EAR-20 application. Peak amplitude and steady-state current were quantified from the glutamate-evoked responses. Percent potentiation was calculated by comparing peak and steady-state amplitudes in control and EAR-20-treated conditions.

Spontaneous activity-dependent NMDAR-mediated excitatory postsynaptic currents (EPSCs) were acquired at 2 kHz and filtered at 1 kHz and measured at a holding potential of −70 mV. EPSCs were measured in 5-min periods in the presence of NBQX + picrotoxin (baseline) and NBQX+ picrotoxin + EAR-20 (100 μM). After the application of EAR-20, 10 μM memantine was added to validate that the EPSCs recorded were NMDAR-mediated. pClamp10/Clampfit10.6 (Molecular Devices) and WinEDR Strathclyde Electrophysiology Software (University of Strathclyde, Glasgow) were used to record, detect and analyze the amplitude, inter-event interval, frequency and charge transfer (as area under the curve; pA*ms) from single EPSCs.

### Single-channel recordings

For single-channel recordings HEK293 cells were maintained and transiently transfected with a total of 0.6 μg of cDNA constructs encoding wild-type GluN1 and GluN2A subunits as well as green fluorescent protein (GFP), at a ratio of 1:2:0.25. The constructs were mixed with 0.5 μL of Lipofectamine 2000 reagent (Invitrogen), following the manufacturer’s instructions and as previously described.

Events were recorded using the outside-out patch-clamp configuration with borosilicate glass pipettes from borosilicate glass with a resistance of 10–15 MΩ when filled with the internal solution (in mM): 140 CsCl, 5 EGTA, 4 Na_2_ATP, 0.1 Na_3_GTP and 10 HEPES, adjusted to pH 7.35 with CsOH. The external solution contained (in mM): 140 NaCl, 5 KCl, 1 CaCl_2_, 10 glucose, and 10 HEPES adjusted to pH 7.40 with NaOH. Currents were activated by 1 mM glutamate and 50 μM glycine, obtained at a holding voltage of −80 mV and digitized at 40 kHz with low-pass filtering at 10 kHz by means of Axopatch 200B amplifier, Digidata 1440A interface and pClamp10 software (Molecular Devices Corporation). To evaluate the effect of EAR-20, 100 µM of the peptide was applied to the bath after baseline currents were recorded in the presence of glutamate and glycine.

Single-channel analysis was performed using WinEDR Strathclyde Electrophysiology Software (John Dempster, University of Strathclyde, UK). Open channel amplitudes were calculated from all-point amplitude histograms and used to calculate single-channel conductance. Open-channel probability (NPo) was calculated as: NPo= (A_1_ + 2A_2_ + 3A_3_ + … + NA_N_)/(A_0_ + A_1_ + A_2_ + A_3_ + …+A_N_) where A_0_ is the area under the curve of amplitude histograms corresponding to current in the closed state, and A_1_ … A_N_ represents the histograms area reflecting the different open-state current levels for 1 to N channels present in the patch. Histogram parameters were obtained from multiple least squares Gaussian fits of the data. Each individual patch contained, on average, 38,122 events for glutamate and 49,628 events for EAR-20 peptide. Records originating from patches containing two or more active channels were considered for further analysis. Open-time and closed-time analyses were performed using idealization methods fitted with WinEDR, which uses a 50% detection threshold to set the positions of the zero-current level, single-channel current level, and transition threshold. Open and closed times were fitted to exponential distributions using maximum likelihood estimation, and histograms were plotted on a logarithmic time scale. Dwell-time distributions were best fit with two open and four closed states. For comparison, control recordings without EAR-20 were done. The peptide effect was assessed by comparing the open probability (NP_o_) and the time constants of individual open and closed states before and after EAR-20 application.

### Statistical analysis

Comparisons between experimental groups were evaluated using Prism (GraphPad Software, Inc.). Data were first tested for normality using the Shapiro-Wilk test. For single comparisons, either Student’s t-test (for parametric data) or Mann-Whitney U-test (for non-parametric data) were used. For comparing paired non-parametric groups, Wilcoxon matched-pairs signed rank test was used. For multiple group comparisons, in case of parametric data, a One-Way Analysis of Variance (ANOVA) was applied followed by Tukey’s multiple comparisons test; for non-parametric data, the Kruskal–Wallis test was used followed by Dunn’s multiple comparisons test. Data are presented as the means ± SEM from at least three independent experiments.

## Results

### EAR-20 alone is able to elicit NMDAR currents in the absence of natural agonists

**FIGURE 1:**
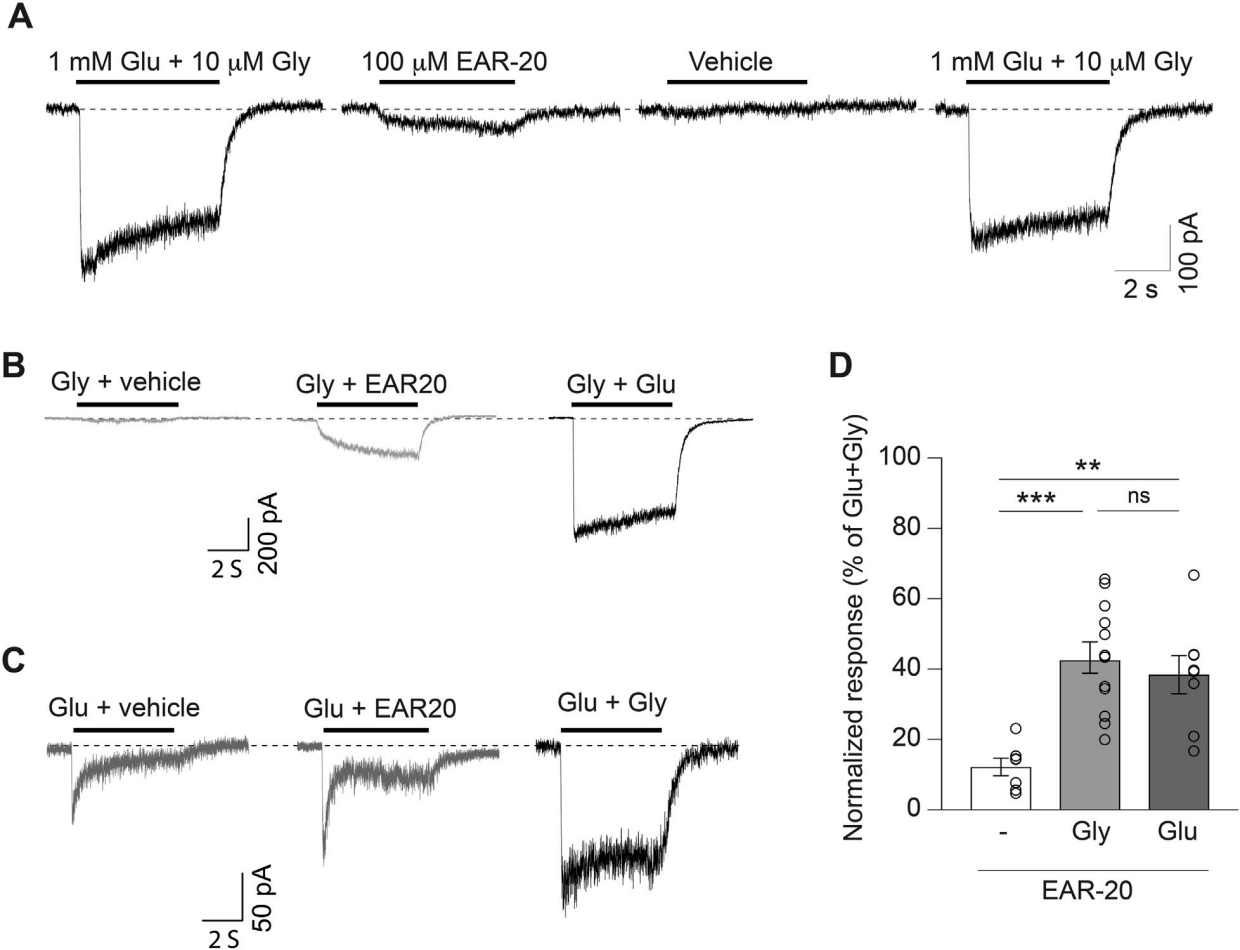
EAR-20 peptide elicits NMDAR-mediated currents in the absence of one or both natural agonists. **(A)** Representative whole-cell patch-clamp recording from a HEK 293T cell expressing GluN1/GluN2B receptors. Fast application of 1 mM glutamate + 10 μM glycine (first trace) evokes a robust NMDAR current. The second trace shows a small current elicited by 100 μM EAR-20 alone; the third trace corresponds to vehicle application (control physiological solution) and the fourth shows a repeat application of glutamate + glycine in the same cell. **(B)** Example recordings in the same conditions as in A showing responses to 10 μM glycine applied with EAR-20 (middle trace), 10 μM glycine alone (right), or 1 mM glutamate + 10 μM glycine (left). **(C)** Responses in the same conditions as in A and B for 1 mM glutamate applied with EAR-20 (middle trace), glutamate alone (right), or 1 mM glutamate + 10 μM glycine as control (left). **(D)** Quantification of responses normalized to maximum current at the steady state elicited by 1 mM glutamate + 10 μM glycine. Bars represent the mean ± SEM for EAR-20 alone (white bar), EAR-20 + glycine (light gray) and EAR-20 + glutamate (dark gray). Open circles represent individual cell data points. **p < 0.01, ***p < 0.001, ns: not significant (ANOVA followed by Tukey’s *post hoc* test).

EAR-20 is a randomly generated peptide synthesized as a control as part of a study evaluating a series of structurally modified peptides derived from the Con-G sequence, aimed at selectively inhibiting NMDA receptors containing the GluN2B subunit. Importantly, EAR-20 is not a direct scrambled version of the native Con-G peptide, but rather a scrambled sequence of one of its modified derivatives (Reyes-Montaño et al., 2023). However, EAR-20 demonstrated the ability to mildly activate specific currents in cultured hippocampal neurons, presumably by acting on NMDARs. Due to this observation, we decided to evaluate the effect of the peptide on NMDA-type glutamate currents. To validate these initial observations, we measured the activity of EAR-20 in HEK-293T cells transfected with di-heteromeric GluN1-GluN2B receptors in the whole-cell configuration of the patch clamp technique. As expected, cells transiently expressing NMDARs responded to the fast application of 1 mM glutamate and 10 μM glycine with a rapid peak activation of the current followed by a steady-state current for the 5-s duration that agonists application lasted (Figure 1A; first trace). In the absence of the endogenous co-agonists, EAR-20 application at 100 µM elicited a small activation of GluN1-GluN2B receptors (Figure 1A; second trace) compared with the application of extracellular solution where no current signal was observed (Figure 1A, third trace). The maximal agonist activity of EAR-20 was 12.18% ± 6.9% (n = 6 cells; Figure 1D), compared to the glutamate plus glycine application effect. We observed the same agonistic activity when it was applied to GluN1-GluN2A receptors (13.31% ± 6.5%; n = 3; data not shown).

After this striking observation, we wondered whether the binding of EAR-20 to the NMDAR affects the requirement for simultaneous glutamate and glycine binding for receptor activation and whether EAR-20 could potentiate NMDAR currents in the presence of only one of their natural agonists, either glutamate or glycine. We evaluated responses to glycine or glutamate alone in the presence of EAR-20 on GluN1-GluN2B receptors in the same experimental conditions (Figures 1B,C, respectively). In these experiments, the current response elicited in the NMDAR by its natural co-agonists–glycine (10 µM) and glutamate (1 mM) – was defined as 100%. The response generated by EAR-20 in the presence of glycine or glutamate was expressed as a percentage of this value. Application of only glycine plus EAR-20 resulted in responses of 43.22% ± 18.1% (Figure 1B; second trace). As happened with the application of EAR-20, the maximum current generated by the application of glycine plus EAR-20 took a few seconds to reach a stable state, hence, we measured the current at steady state and compared it with the maximum current elicited by glutamate plus glycine at the same time period (Figure 1D). The response generated by EAR-20 in the presence of glutamate was 38.33% ± 17.1% at steady-state, but under these conditions, we observed a clear peak response, which was not observed in the EAR-20 plus glycine application. Interestingly, while the application of glycine alone did not result in any measurable response (Figure 1B; grey; first trace), the application of glutamate alone (Figure 1C; dark grey; first trace) generated a significant current response relative to the maximum current elicited by Glu plus Gly, which was evident at the peak and remained significant at steady-state at the end of the application. However, in the presence of EAR-20 the steady state current tends always to increase during the application period. Despite the fact that simultaneous binding of glutamate and glycine (or serine) is necessary for the efficient activation of NMDARs, glutamate alone has been previously shown to slightly activate the channel, although much less efficiently (Johnson and Ascher, 1987). The observed response to glutamate could result from low trace levels of contaminating glycine in the recording solution (*see Discussion*). Together, these results indicate that EAR-20 has an intrinsic ability to activate NMDA receptors to a significant degree, and that the requirement for simultaneous glutamate and glycine co-agonist binding for receptor activation could be modified by EAR-20.

### EAR-20 is a positive allosteric modulator with differential effects on GluN1-GluN2 receptor subtypes

Since the EAR-20 peptide can potentiate NMDAR currents when applied solely with one of the agonists (Glu or Gly), we suspected that it might act as a positive allosteric modulator facilitating the gating of NMDARs. Another significant piece of evidence that EAR-20 could display PAM activity came from previous experiments testing its agonist activity where we observed that after EAR-20 had been applied to the bath at 500 μM, the first subsequent NMDAR response to Glu and Gly was increased during this initial application (*data not shown*). A similar effect was observed when EAR-20 was applied alone after NMDAR stimulation with its natural agonists: the first EAR-20 application elicited a higher response compared to subsequent applications. These effects might be explained by an incomplete washout of either EAR-20 before the application of both agonists or incomplete removal of agonists before EAR-20 application (*see* Figure 1D *where EAR-20 effect is potentiated in the presence of glycine or glutamate*) suggesting that EAR-20, in combination with glutamate and glycine, could act as a potentiator of NMDAR currents. This led us to test whether EAR-20 might behave as a PAM of NMDARs.

**FIGURE 2:**
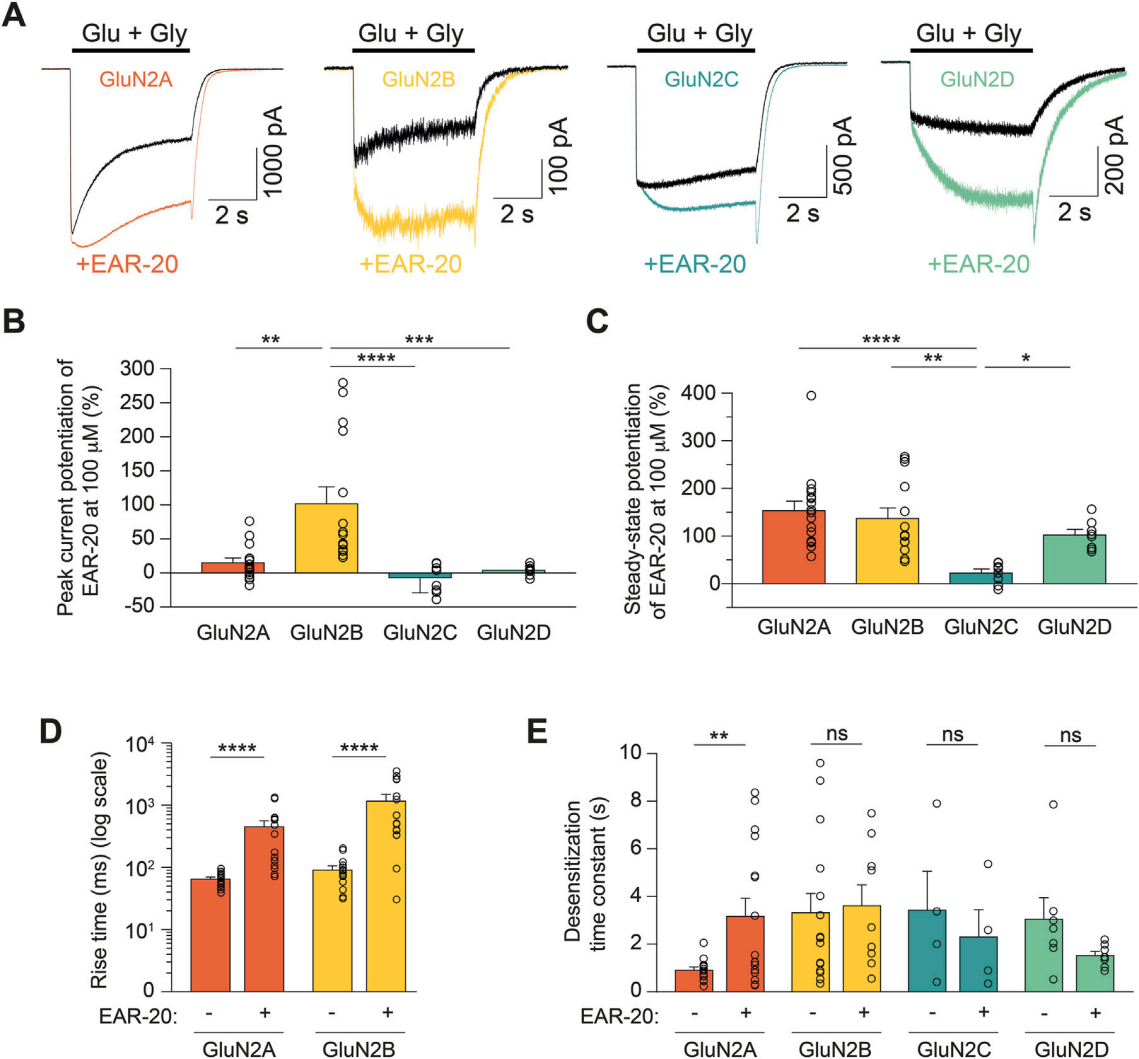
EAR-20 acts as a positive allosteric modulator of NMDARs and affects NMDAR kinetics in a subunit-dependent manner. **(A)** Representative whole-cell patch-clamp recordings from HEK-293T cells expressing recombinant diheteromeric GluN1/GluN2A, GluN1/GluN2B, GluN1/GluN2C or GluN1/GluN2D NMDARs. Cells were rapidly activated by 1 mM glutamate and 50 µM glycine (black traces), and responses in the presence of 100 µM EAR-20 are shown in color for each NMDAR subtype (orange, yellow, blue and green, respectively). **(B)** Quantification of peak current potentiation by 100 µM EAR-20, expressed as percentage of control peak (elicited by glutamate and glycine alone alone). Data is shown as mean ± SEM; individual recordings are represented as open circles. Statistical analysis was performed using Kruskal–Wallis test followed by Dunn’s *post hoc* tests. Significance is as follows: p < 0.05 (*), p < 0.01 (**), p < 0.001 (***), and p < 0.0001 (****). **(C)** Quantification EAR-20 potentiation of the steady-state current for the same NMDAR subtypes as in **B**. Data and statistical analysis as described in **B**. **(D)** Quantification of recordings where the time to reach the maximum current response (in milliseconds) upon fast agonist application (1 mM glutamate and 50 μM glycine), measured in the absence (−) and presence (+) of 100 μM EAR-20. Note that the y-axis is shown in logarithmic scale. Statistical significance was assessed by Mann Whitney U-test for both GluN2A and GluN2B (****p < 0.0001). **(E)** Weighted desensitization time constants (in seconds) extracted from double exponential fits of current traces from diheteromeric NMDAR combinations. EAR-20 significantly reduced desensitization time in GluN2A-containing receptors only. Student’s t-test for all paired groups (**p < 0.01).

To evaluate the potential PAM activity and NMDAR subtype specificity of EAR-20, we expressed different di-heteromeric recombinant NMDARs (GluN1-GluN2A, GluN1-GluN2B, GluN1-GluN2C or GluN1-GluN2D) and activated their mediated currents by rapidly applying endogenous coagonists (1 mM glutamate and 50 μM glycine) in the absence or presence of 100 μM EAR-20. Potentiation percentages elicited by EAR-20 were quantified relative to currents generated by glutamate and glycine (set as 0%) either at the peak–within the first 50 milliseconds following agonist application–or at the steady state–between 3 and 4 s after application. We found that EAR-20 application increased the currents generated by glutamate and glycine, especially a few seconds after application, during the steady state of the elicited currents for all combinations tested (Figure 2A). At the end of the agonist pulse, a transient increase in current was frequently observed in EAR-20 condition, which then decreased to baseline levels within a few milliseconds after the cell was again bathed into the control solution (see Discussion).

Figure 2B shows that EAR-20 produced a significant instantaneous potentiation (peak current) only for GluN1-GluN2A and GluN1-GluN2B with this rapid effect being more pronounced in GluN2B (15.68% ± 6.19% and 102.6% ± 24.02% for GluN2A and GluN2B; p = 0.023 and p < 0.0001, respectively). In contrast, no significant peak effect was observed for the other combinations (−8.04% ± 7.48% and 4.97% ± 2.63% for GluN2C and GluN2D, respectively; p = 0.3185 and p = 0.1011, respectively). When comparing across different combinations, the increase in peak current elicited by EAR-20 was significantly greater for GluN1-GluN2B compared to all other conditions (p = 0.0022, p < 0.0001, and p = 0.0009 vs. GluN1-GluN2A, GluN1-GluN2C, and GluN1-GluN2D; Kruskal–Wallis test with multiple comparisons).

In contrast to the clear peak effect observed only for GluN2B-containing NMDARs, EAR-20 potentiated the steady-state currents of all combinations, albeit to varying degrees. EAR-20 produced a maximum current potentiation for the di-heteromers GluN1-GluN2A and GluN1-GluN2B of 154.30% ± 19.0% and 137.9% ± 21.0% respectively (Figure 2C). The PAM activity over the less frequently expressed subunits, GluN2C and GluN2D, was lower than on the more widely expressed subunits, GluN2A and GluN2B. Although no significant differences were found for GluN2D (102.45% ± 11.1%) compared to GluN2A or GluN2B, currents on di-heteromeric GluN2C NMDARs were less potentiated by EAR-20 (23.31% ± 7.6%; Kruskal-Wallis-test, p < 0.0001, p = 0.0010 and p = 0.042 vs. GluN2A, GluN2B and GluN2D, respectively; Figure 2C).

### EAR-20 differentially affects NMDAR kinetics depending on the GluN2 subunit composition

Despite the described differential effect on the peak and steady-state currents, in all tested conditions, EAR-20 appeared to reduce NMDAR desensitization over time, an effect that developed gradually over the following milliseconds after the cell’s exposure to EAR-20 (Figure 2A) in all combinations. That fact prompted us to characterize the effect of the peptide on NMDAR kinetics. Hence, based on our electrophysiological recordings, we calculated the rise time and desensitization rate in the absence and presence of EAR-20 for all the di-heteromeric NMDAR combinations.

We calculated the rise time as the time interval between the initial onset of the current following compounds application and the point in which evoked current reached its peak. A high degree of variability was observed in the time required to reach the maximum peak current across recordings, regardless of the studied condition. However, in recordings for GluN2C- and GluN2D-containing receptors the current did not show a clear peak and instead the current continued to develop gradually over several seconds. Therefore, rise time was analyzed only in GluN2A- and GluN2B-containing receptors, which, in fact, were the most affected subunits by EAR-20. The rise time was significantly increased for GluN2A-containing NMDARs (65.85 ± 4.03 ms vs. 460.3 ± 101.4 ms for agonists and agonists+EAR-20, respectively; ****p < 0.0001; Figure 2D). Similarly, the rise time of GluN2B-containing NMDAR di-heteromeric receptors was increased in responses with EAR-20 present in the bath (92.67 ± 13.06 ms vs. 1,190 ± 306 ms for agonists and agonists+EAR-20, respectively; ****p < 0.0001; Figure 2D).

When we compared the effect of EAR-20 on the desensitization kinetics of different NMDAR combinations, only NMDARs formed by GluN1/GluN2A–which is the combination that displays the faster kinetics–showed an altered desensitization time due to EAR-20 (0.92 ± 0.11 s vs. 3.18 ± 0.73 s; **p = 0.0063; Figure 2E). In contrast, the other di-heteromeric combinations, which normally have lower desensitization rates, showed no differences between control traces activated solely by glutamate and glycine and those activated with agonists plus EAR-20.

### Dose–response relationship of EAR-20 at GluN2A- and GluN2B-containing NMDARs

**FIGURE 3:**
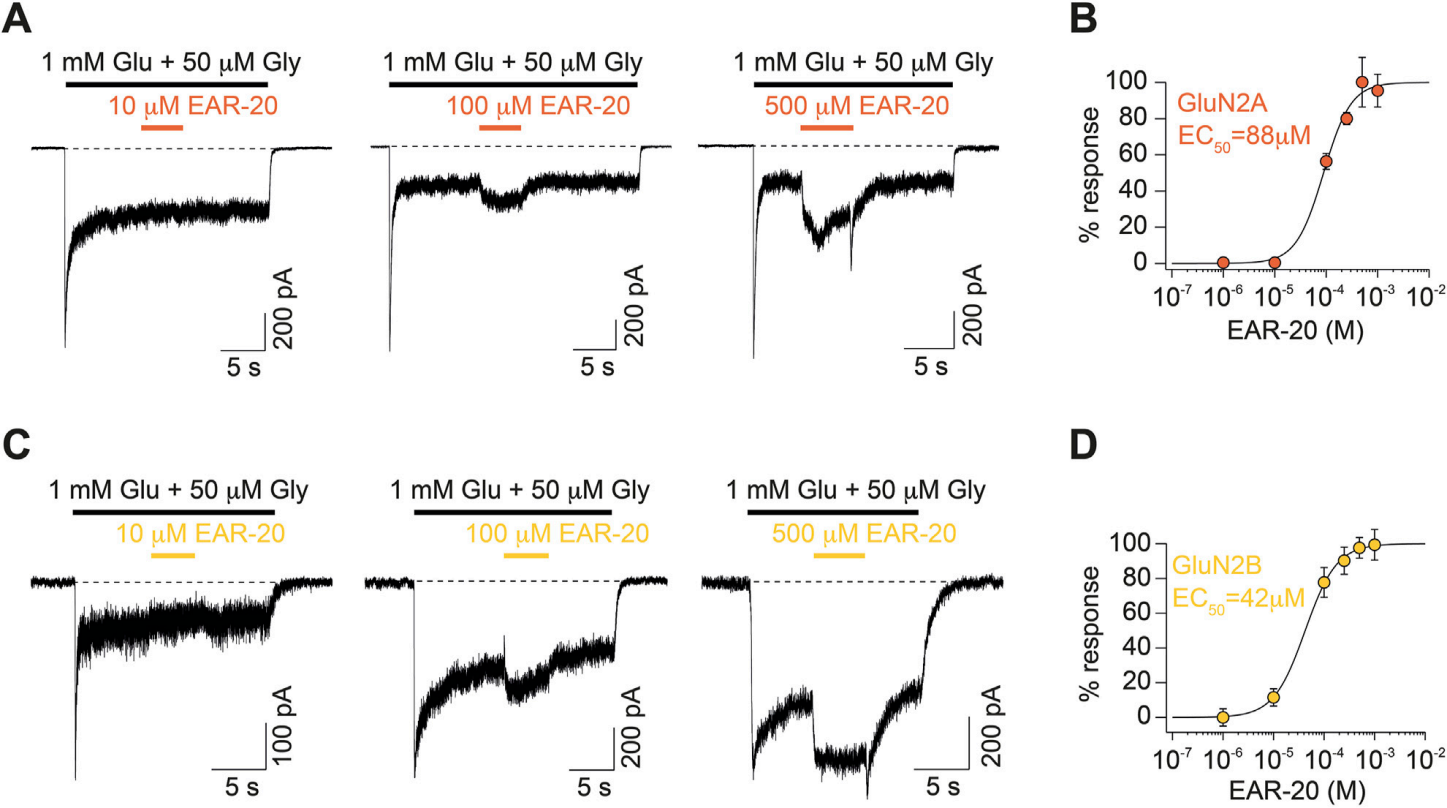
EAR-20 dose-dependently potentiates NMDAR-mediated currents. **(A)** Representative whole-cell patch-clamp recordings from HEK-293T cells expressing recombinant GluN1/GluN2A NMDARs. Currents were evoked by fast application of 1 mM glutamate and 10 μM glycine and increasing concentrations of EAR-20 were applied onto the steady-state current using a 5-s puff delivered with a microinjector. Traces show responses to 10 μM, 100 μM, and 500 μM EAR-20. **(B)** Concentration-response curve for EAR-20 potentiation of GluN2A-containing receptors, expressed as percentage of current potentiation relative to control responses (agonists alone). Data points represent mean ± SEM; n = 5-7 cells per condition. EC_50_ value of 88 μM was calculated using a sigmoidal fitting to the Hill equation. **(C)** Same as in A but for GluN2B-containing NMDARs. **(D)** Same as in B but for GluN2B-containing NMDARs. n = 4-7 cells per condition The Hill fit yielded an EC_50_ values of 42 μM for GluN2B.

We next determined the dose-response relationship for EAR-20 in HEK-293T cell expressing recombinant GluN1-GluN2A and GluN1-GluN2B NMDARs, as these were the two subtypes that exhibited the greatest effect of EAR-20. After reaching a stable steady-state response under saturating agonist conditions (1 mM Glu and 50 µM Gly), we applied EAR-20 at different concentrations (Figures 3A,C). Due to the limited availability of EAR-20 compound we applied it using a microinjector in a solution containing the same agonist concentrations. The EC_50_ for EAR-20 was similar for GluN1-GluN2A and GluN1-GluN2B within the same range: 88.74 and 42.23 µM, respectively (Figures 3B,D). However, EAR-20 showed slightly higher potency at NMDARs containing GluN2B, as lower concentrations of the peptide produced greater current activation, as shown in Figures 3A,C (third traces).

These results indicate that EAR-20 functional binding to the NMDAR is strongly determined by receptor’s subunit composition. Specifically, the binding process appears to be favored in di-heteromeric receptors containing GluN2B subunits and to a lesser extent, in those incorporating GluN2A. Consequently, these subunits exert a greater impact on receptor gating modulation by EAR-20. This differential sensitivity is not unexpected, as NMDAR subunit composition is known to influence receptor pharmacology, with classic examples including ifenprodil’s selectivity for GluN2B-containing receptors and Zn^2+^ inhibition preferentially affecting GluN2A-containing receptors (Burger et al., 2012; Zhu and Paoletti, 2015).

### EAR-20 favors the open states of the NMDA receptor

**FIGURE 4:**
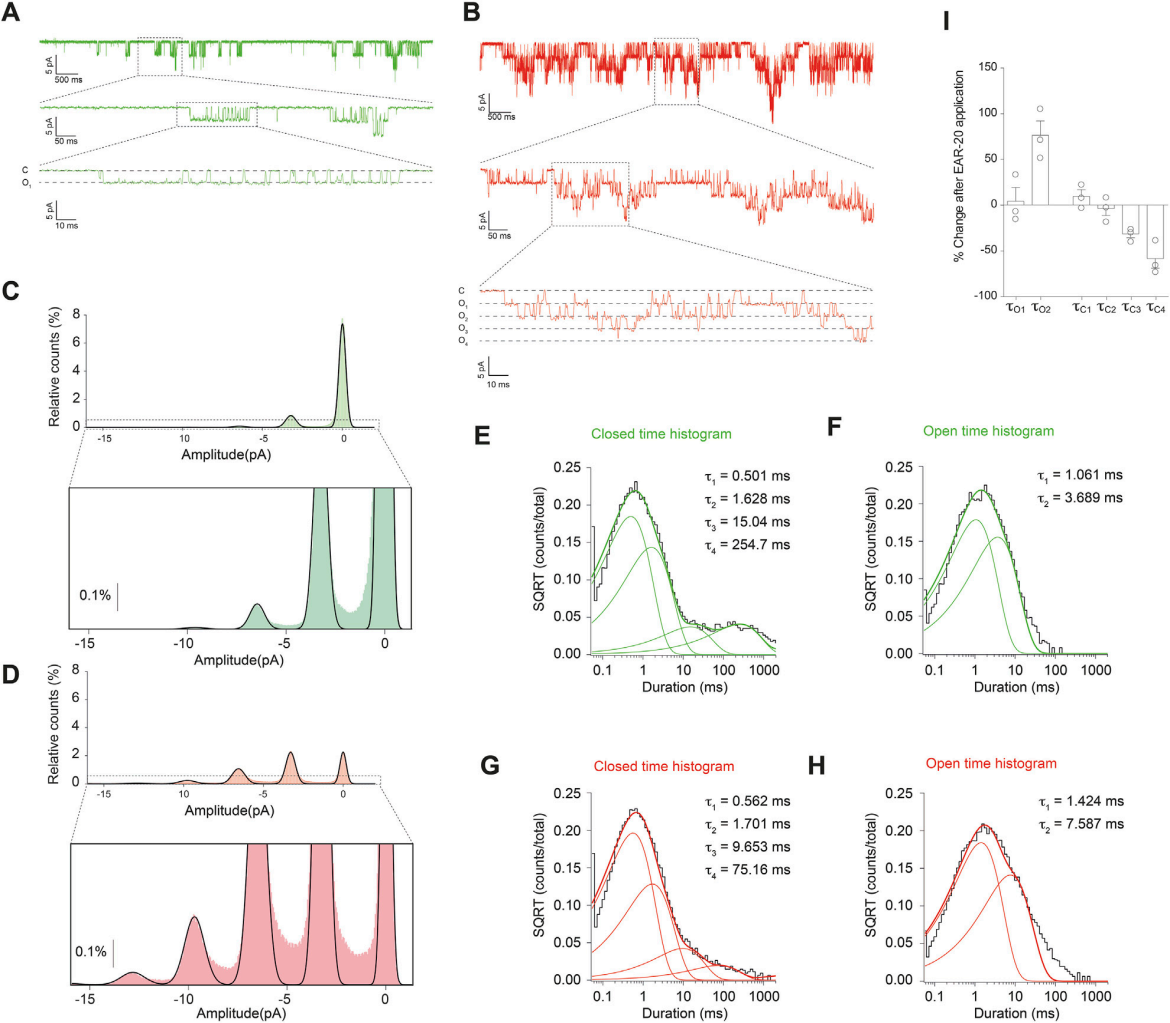
EAR-20 alters single-channel kinetics of GluN1/GluN2A receptors. **(A,B)** Representative current traces from an outside-out patch of wild type GluN1/GluN2A receptor activity in HEK-293T cells before **(A)** and after **(B)** application of 100 µM EAR-20 in the continuous presence of 1 mM glutamate and 50 µM glycine. **(C,D)** Amplitude histogram of events detected in the absence (C; green) or the presence (D; red) of EAR-20 with magnification of boxed regions. EAR-20 increases the frequency of higher-amplitude events, as reflected in taller Gaussian peaks, while the peak positions remain unchanged indicating that unitary current amplitude (and thus single-channel conductance) is not affected by EAR-20. **(E)** Dwell-time histogram from the closed interval durations with superimposed distributions (16,361 events) of the example control agonists conditions recording shown in A** (F)** Open dwell-time histograms from the same recording fitted with the sum of four exponential components (16,361 events). **(G,H)** Closed and open dwell time histograms for EAR-20 recording shown in B (23,206 events each). EAR-20 increases the duration of the longer open component and reduces the lifetime of long closed components, consistent with enhanced channel gating and reduced desensitization. **(I)** Cumulative results (mean ± SEM) show that EAR-20 addition increases the durations of open the τ_2_ time component and the duration of τ_3_ and τ_4_ long closed components (p < 0.05 for all three components; paired Student’s t-test).

To gain insight into the mechanisms by which EAR-20 exerts its potentiating effect, we studied single-channel currents in the outside-out configuration. This set up allowed us to activate NMDARs with their natural agonists, glutamate and glycine, prior to the application of EAR-20 to the bath. Single-channel data obtained from GluN1/GluN2A-transfected cells activated by glutamate and glycine (Figure 4A) showed a significant increase in channel activity after the addition of EAR-20 to the bath (Figure 4B). Figures 4C,D show the frequency histograms before (green) and after (red) bath application of EAR-20 at 100 µM. While in baseline conditions the channel is closed the majority of the time (peak at 0 pA), in the presence of EAR-20, the peaks corresponding to one, two, three or four open channels are significantly larger, as evidenced by open probability (NPo), that was increased nearly 5-fold (0.15 ± 0.01 vs. 0.72 ± 0.08 for agonists and agonists+EAR-20, respectively). This agrees with data from macroscopic currents. We then analyzed the open and closed times for both control and EAR-20. Our recordings were best fitted to a scheme with two open and four distinct closed states as previously reported for GluN1-GluN2A (Erreger and Traynelis, 2008). The analysis of closed times revealed a reduction in the time spent in conformational states τ_3_ and τ_4_ under the EAR-20 condition (12.9 ± 1.1 ms vs. 8.9 ± 0.4 ms for control and EAR-20 in closed state 3 and 248.5 ± 7.3 ms vs. 102.7 ± 21.2 ms for control and EAR-2 in closed state 4) representing a decrease of 31% and 59% respectively (Figure 4I). These prolonged closed periods have been associated with increased receptor desensitization (Cummings and Popescu, 2015), suggesting that EAR-20 may reduce desensitization by decreasing occupancy of these closed states. In contrast, open state 2 clearly shifted toward longer open times (3.8 ± 0.1 ms vs. 6.7 ± 0.5 ms for control and EAR-20; 76% increase) with open state 1 showing a more modest increase of 11%. The increase in open probability (Figures 4C,D) observed with EAR-20 could be explained by the decrease in closed times (Figures 4E,G) together with the increase in open times (Figures 4F,H) in the receptor.

### EAR-20 enhances native NMDA receptor function in hippocampal neurons

**FIGURE 5:**
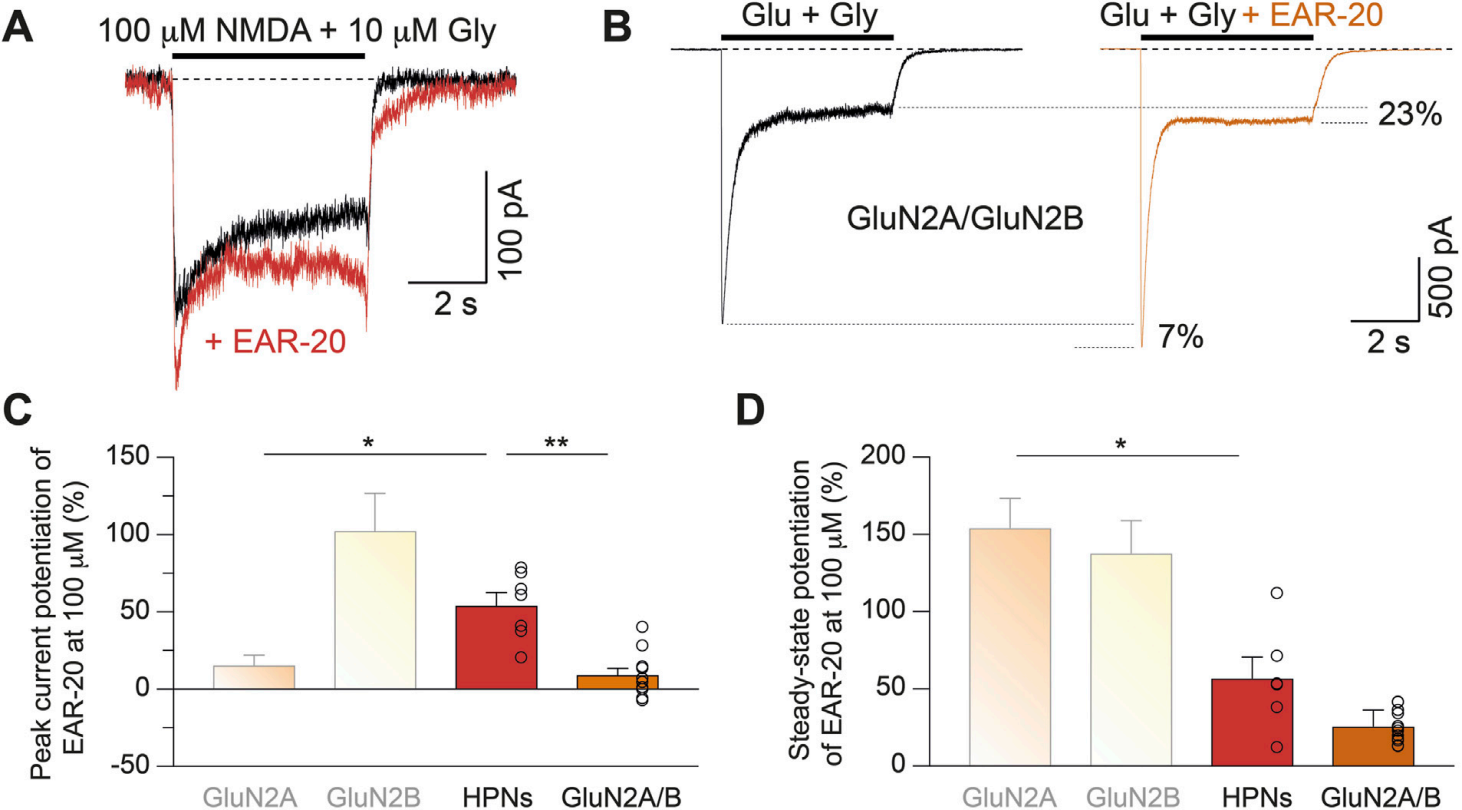
EAR-20 enhances native NMDAR function and reveals limited potentiation in triheteromeric GluN2A/GluN2B-containing receptors. **(A)** Representative whole-cell patch-clamp recording from a cultured hippocampal neurons (HPN) showing NMDA receptor-mediated currents evoked by rapid application of 1 mM glutamate and 10 µM glycine, either alone (black trace) or co-applied with 100 µM EAR-20 (red trace). **(B)** Traces from a HEK-293T cell expressing recombinant tri-heteromeric GluN1/GluN2A/GluN2B receptors activated with glutamate and glycine alone (black trace) or together with EAR-20 (orange trace). Due to lower enhancement compared diheteromeric receptors, traces are shown separately for clarification. **(C)** Quantification of peak current potentiation (mean ± SEM) by 100 µM EAR-20 in HEK-293T cells expressing GluN1/GluN2A/GluN2B tri-heteromers (red), native receptors from HPNs (orange) and recombinant diheteromeric GluN1/GluN2A and GluN1/GluN2B receptors (in degraded colors; data from Figure 2), included for comparison. Bars represent mean ± SEM with individual values denoted as open circles. **(D)** Quantification of steady-state current potentiation under the same conditions as in C. While EAR-20 robustly enhanced GluN2A- and GluN2B-containing di-heteromers (in degraded colors; data from Figure 2), its effect was markedly reduced in triheteromeric receptors and native hippocampal neurons.

Once determined the effect of EAR-20 on NMDAR currents in expression systems, we decided to evaluate it on native NMDARs. Hence, we performed whole-cell patch-clamp recordings from cultured hippocampal neurons, which putatively express GluN2A and GluN2B subunits, with a solution-switching system by rapidly applying agonists or agonists plus 100 µM EAR-20 onto the Soma of hippocampal neurons. Figure 5A shows that the application of EAR-20 (trace in red) increased hippocampal currents mediated by NMDARs, as evidenced by an increase in both peak and steady-state currents (quantified in Figures 5C,D, respectively), thus confirming the PAM effect of EAR-20 on native NMDARs. The peak response potentiation induced by EAR-20 was intermediate between GluN2A and GluN2B conditions recorded previously in HEK293 cells (54.26% ± 8.15% for native NMDARs *versus* 15.68% ± 6.19% for GluN2A-containing heterologous NMDARs or 102.6% ± 24.02% for GluN2B-containing heterologous NMDARs). However, the percentage of potentiation of EAR-20 at the steady-state in neurons was 56.9%, which was considerably lower than those observed for GluN1-GluN2A and GluN1-GluN2B (154.3% and 137.9%, respectively). These differences in the PAM activity of EAR-20 in HEK 293T cells transfected (either with GluN1-GluN2A or GluN1-GluN2B) and hippocampal neurons could be attributed to the presence of a larger population of NMDARs assembled as GluN1-GluN2A-GluN2B tri-heteromeric receptors in differentiated hippocampal neurons of neonatal and adult mice (10 DIV). Indeed, this population has been reported to represent up to one-third of the total NMDARs in the adult hippocampus, constituting an important component of glutamatergic synapses in CA1 and CA3 (Rauner and Köhr, 2011).

Given that tri-heterotetramers display distinct biophysical and pharmacological properties compared to GluN2A or GluN2B di-heteromeric NMDARs (Hansen et al., 2014), and to better understand the responses observed in hippocampal neuron assays, we investigated the PAM activity of EAR-20 on tri-heteromers expressed in HEK 293 cells. To ensure the exclusive surface expression of this class of NMDARs, we employed constructs incorporating dual retention signals (Stroebel et al., 2014). Briefly, these retention signals allow the selective trafficking of tri-heteromeric NMDARs by retaining di-heteromeric receptors in the endoplasmic reticulum, thereby exclusively ensuring GluN2A/GluN2B-containing receptors at the cell surface. We found that the effect of EAR-20 on tri-heteromers displayed a different profile compared to di-heteromeric combinations. Although the peak potentiation of EAR-20 in GluN2A/2B receptors was similar to that observed in GluN2A-only containing receptors (9.33% ± 3.95% vs. 15.68% ± 6.16% for GluN2A/2B and GluN2A respectively; Figure 5C), the steady state potentiation was only 25.84% ± 3.0% (Figure 5D; orange bar), significantly lower than that observed for di-heteromers GluN1-GluN2A or GluN1-GluN2B (Figure 5D; light orange and pale-yellow bars. Data from experiments in Figures 2C,D for di-heteromeric combinations are used in Figures 5C,D for comparison purposes). Considering the potentiation profiles observed for di-heteromeric and tri-heteromeric NMDARs, the responses measured in hippocampal neurons suggest the presence of a mixed population of receptors as discussed later.

**FIGURE 6:**
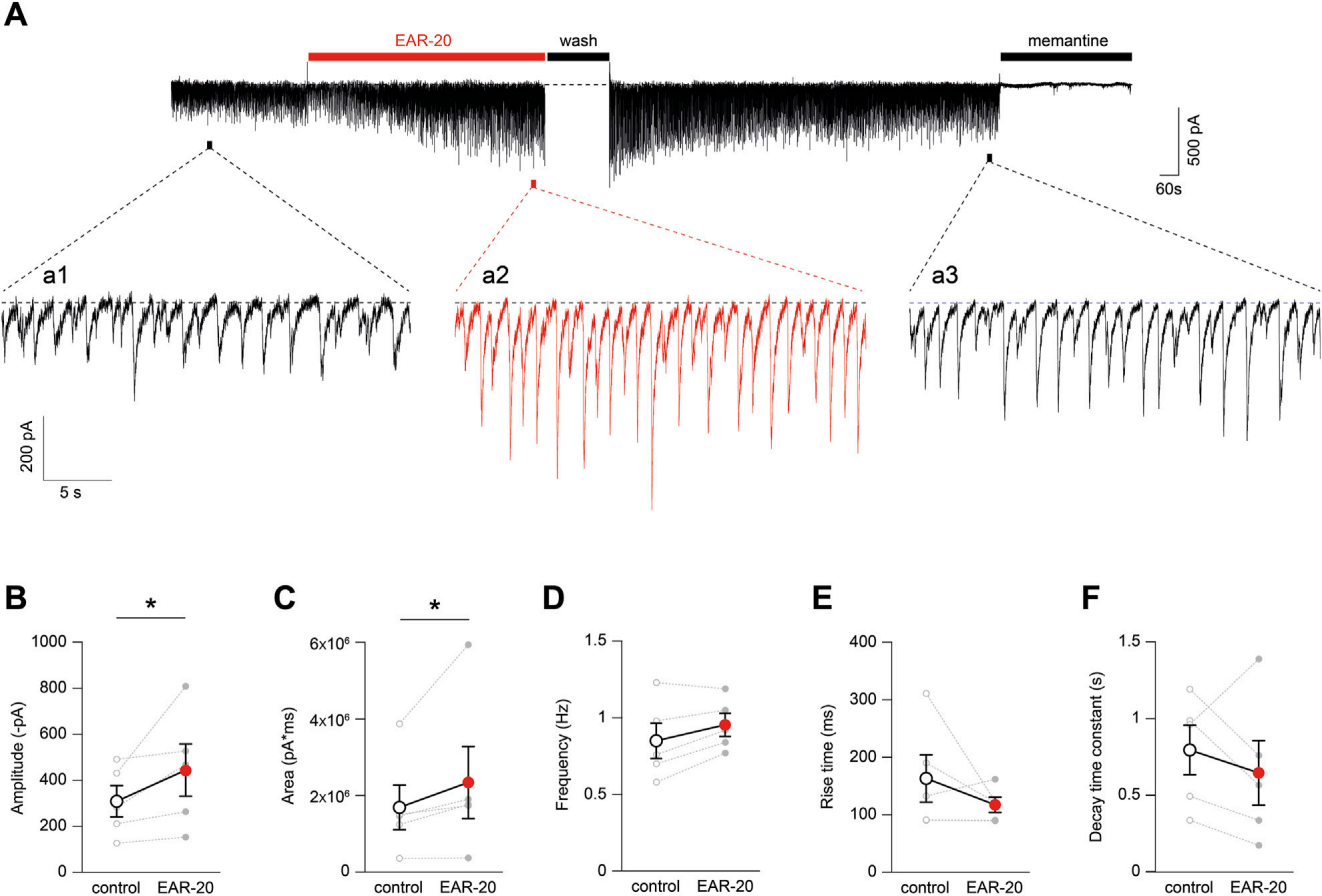
EAR-20 enhances synaptic NMDAR-mediated currents in hippocampal neurons. **(A)** Representative voltage-clamp recording from a hippocampal pyramidal neuron (HPN) showing spontaneous NMDAR-mediated excitatory postsynaptic currents (EPSCs) in control conditions, during bath application of 100 μM EAR-20 (red bar), and after washout. Insets (a1–a3) correspond to expanded traces from the indicated time windows and illustrate the reversible increase in EPSC amplitude induced by EAR-20. **(B)** Quantification of EPSC amplitude before and after 10 min of EAR-20 application. Data are shown as mean ± SEM (large circles) with individual paired values represented as small circles (open and filled for before and during EAR-20 application). *p < 0.05; Wilcoxon matched-pairs signed rank test. **(C)** Area under the curve (AUC) of individual events was also significantly increased by EAR-20 (*p < 0.05; Wilcoxon matched-pairs signed rank test). **(D)** Event frequency showed no significant difference, though a mild increasing trend was observed. **(E,F)** Rise time and decay time constant of EPSCs remained unchanged, indicating that EAR-20 potentiates synaptic NMDAR currents without altering their kinetic profile.

Since EAR-20 exhibited a positive allosteric modulation on somatic NMDARs in cultured hippocampal neurons, we next asked whether a similar modulatory effect could also be observed at synaptic sites. To address this, we examined spontaneous excitatory postsynaptic currents (EPSCs) from hippocampal neuronal cultures in the absence and presence of EAR-20, aiming to determine whether synaptic NMDARs activated by spontaneous release on neurotransmitter were also subject to potentiation by EAR-20. The effect of EAR-20 on synaptic transmission became evident within several minutes of application (Figure 6A), as observed in the representative recording shown, where a clear increase in the amplitude of individual EPSC events occurs during EAR-20 application (Figure 6; inset a2 vs. inset a1). This effect was reversible upon washout (inset a3), and EPSCs were subsequently abolished by application of memantine, confirming their NMDAR-mediated nature. Insets correspond to time windows highlighted in the trace in Figure 6A (top). Quantitative analysis revealed a significant increase in EPSC amplitude from 308.9 ± 67.7 pA before EAR-20 application to 444.3 ± 113.6 pA after 10 min of EAR-20 application (p = 0.0312, Wilcoxon matched-pairs signed rank test; Figure 6B). The peak increment was accompanied by a modest but significant enhancement in the area under the curve (1,690 ± 584 nA*ms vs. 2,343 ± 941 nA*ms; p = 0.0312, Wilcoxon matched-pairs signed rank test; Figure 6C). In contrast, no significant changes were observed in event frequency, although a slight increasing trend was noted (Figure 6D). Additionally, kinetic parameters such as rise time and decay time remained unaltered (Figures 6E,F), indicating that EAR-20 selectively enhances synaptic NMDAR responses without affecting the profile of the currents.

### PAM activity of EAR-20 does not require the NTD of NMDARs

Several molecules acting as allosteric modulators of NMDA receptors have been identified to bind the N-terminal domain (NTD) of GluN subunits including polyamines (Mony et al., 2011), ifenprodil (Burger et al., 2012) or Zn^2+^ (Zhu and Paoletti, 2015). The binding of these modulators alters receptor gating by shifting the equilibrium between active and inactive conformational states, thereby modulating channel open probability and desensitization properties (Mony and Paoletti, 2023). For instance, Zn^2+^ binding to the GluN2A NTD increases desensitization (Amico-Ruvio et al., 2011) while polyamines binding has been reported to reduce it (Lerma, 1992). Given the potentiating effects of EAR-20 on NMDAR currents, we suspected that it might exert this effect by interacting directly with the NTD. To test this possibility, we employed an NMDAR variant lacking the N-terminal domain (ΔNTD), allowing us to determine whether the activity of EAR-20 as a positive allosteric modulator (PAM) depends on this extracellular domain or it is instead, mediated through the interaction on alternative sites, such as the ligand-binding or transmembrane domains.

**FIGURE 7:**
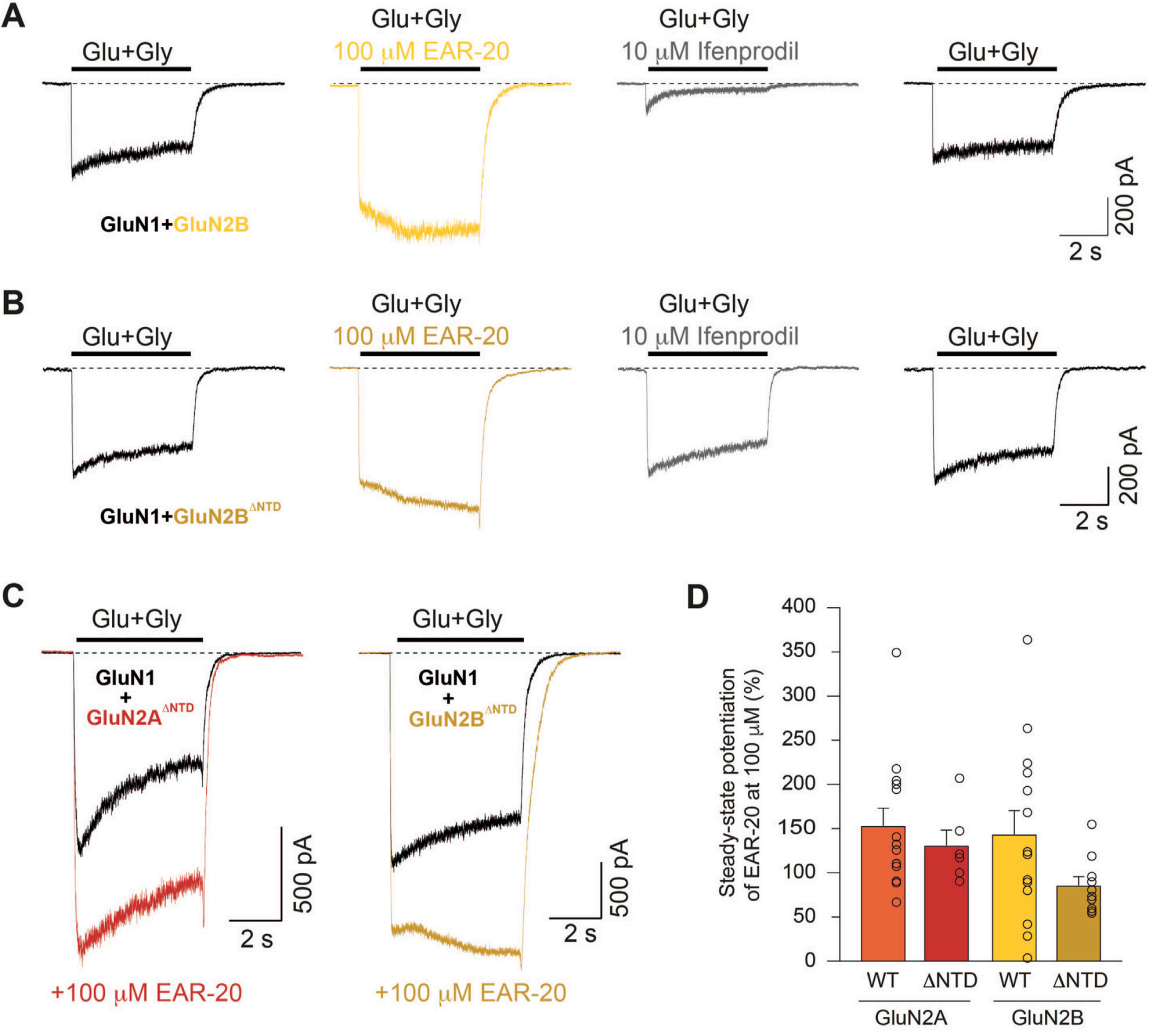
NMDAR N-terminal domain (NTD) is not required for the positive allosteric modulation by EAR-20. **(A,B)** Representative whole-cell patch-clamp recordings from HEK-293T cells expressing either wild-type GluN1/GluN2B **(A)** or NTD-deleted GluN1/GluN2B^ΔNTD^ receptors **(B)**, showing responses to 1 mM glutamate + 10 µM glycine, alone or co-applied with 100 µM EAR-20 or 10 µM ifenprodil. **(C)** Representative traces of EAR-20 modulation of NTD-deleted GluN1/GluN2A and GluN1/GluN2B receptors, showing preserved potentiation. **(D)** Quantification of steady-state current potentiation by 100 µM EAR-20 for wild-type and ΔNTD receptors. Data are shown as mean ± SEM with individual values represented by open circles. Deletion of the NTD does not impair the PAM effect of EAR-20. No statistical difference was found between groups using Kruskal–Wallis.

Thus, to assess whether the NTD of either the GluN1, GluN2A, or GluN2B subunit influences EAR-20 activity, and to further identify a potential binding site of the EAR-20 peptide on the NMDAR, we performed whole-cell patch-clamp recordings in HEK 293T cells transfected with either *wild-*type or NTD-lacking subunits (GluN1^ΔNTD^, GluN2A^ΔNTD^ and GluN2B^ΔNTD^), given that cells transfected with these truncated subunits are capable of assembling functional NMDA receptors (Paoletti et al., 2000; Gielen et al., 2009; Mony and Paoletti, 2023). When we expressed these NTD-lacking subunits in HEK293 cells, all tested combinations gave rise to functional receptors, except those containing GluN1^ΔNTD^ which did not generate currents of sufficient magnitude for reliable analysis. Receptors composed of GluN1-GluN2A^ΔNTD^ and GluN1-GluN2B^ΔNTD^ exhibited response currents similar to those of wild-type receptors containing the NTD as measured by the extent of macroscopic desensitization (ratio of steady-state to peak current; Figure 7D). Since GluN2B NTD has been demonstrated to be essential for the modulation of NMDA receptor function by ifenprodil, we first examined the effect of this compound on wild-type receptors (GluN1-GluN2B; Figure 7A) and on mutant receptors (GluN1-GluN2B^ΔNTD^; Figure 7B). Application of ifenprodil (10 µM) inhibited currents from GluN1/GluN2B receptors about 88.23% ± 0.8% (Figure 7A, third trace). In contrast, no inhibitory effect was observed when ifenprodil was co-applied together with agonists in HEK-293 cells expressing GluN1-GluN2B^ΔNTD^ receptors (Figure 7B; third trace). Given that ifenprodil binds at the NTD interface of GluN1 and GluN2B subunit (Karakas et al., 2011), these results proved that mutant receptors were functionally expressed in HEK 293T cells. Importantly, Figures 7C,D show that EAR-20 at 100 µM was still able to potentiate agonist-evoked responses in both GluN1-GluN2A^ΔNTD^ and GluN1-GluN2B^ΔNTD^ receptors. In GluN1-GluN2A^ΔNTD^ the potentiation was 131.1% ± 17.25%, compared to 153.3% ± 19.9% in the non-truncated receptor (p = 0.7791; Mann-Whitney test). Similarly, in GluN1-GluN2B^ΔNTD^ receptors, potentiation reached 85.82% ± 9.9% *versus* 143.7% ± 26.8% in the wild-type receptor (p = 0.0933; Student’s t-test). Despite some variability, the overall response profiles were comparable suggesting that the NTD is not required for the PAM on NMDARs by EAR-20 (Figure 7D).

### Identification of a potential EAR-20 binding site on the NMDAR

To further investigate the potential binding site of EAR-20 on the NMDA receptor, we performed molecular modelling and docking strategy to predict potential receptor interaction sites. Structural modeling of EAR-20 was performed to obtain a three-dimensional conformation of the peptide before docking simulations on the GluN1-GluN2B di-heteromer to identify key residues potentially involved in EAR-20 binding and to guide subsequent mutagenesis experiments to validate functional interactions.

**FIGURE 8:**
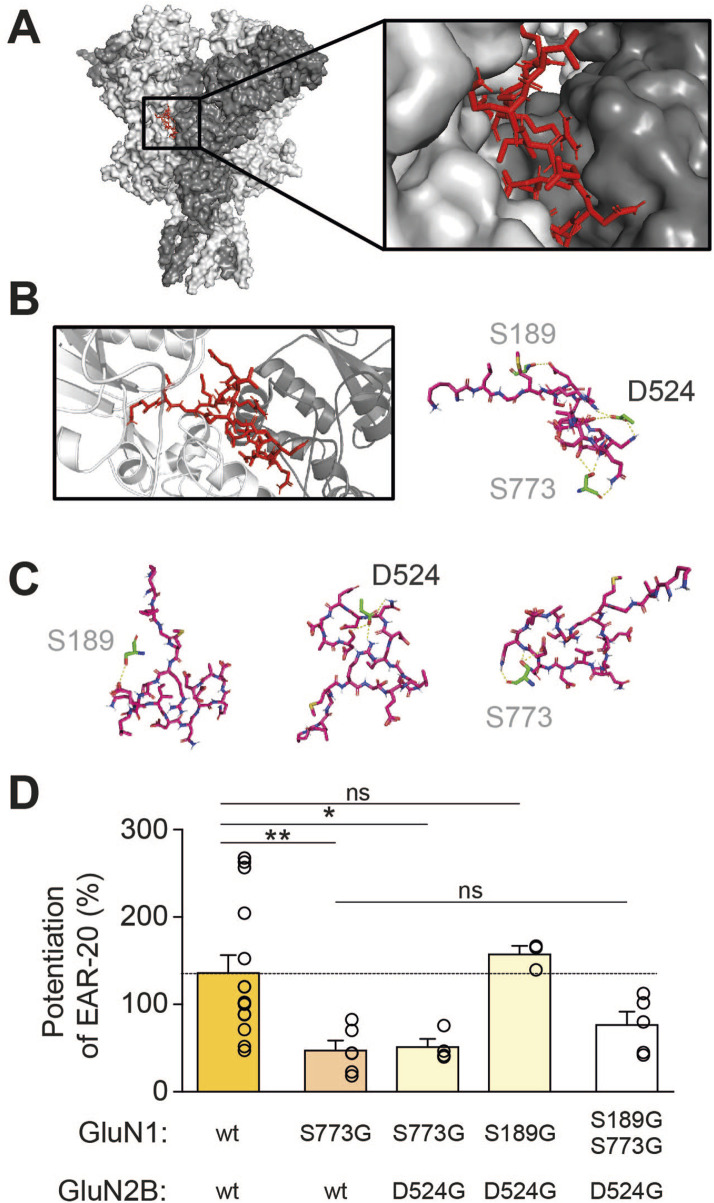
Molecular docking reveals a critical role for GluN1 serine 773 in the modulatory effect of EAR-20. **(A)** Molecular docking simulation shows EAR-20 secondary structure predicted by PEP-FOLD4 and validated by Ramachandran analysis (in red) binding at the dimer interface between GluN1 (light grey) and GluN2B (dark grey) subunits of NMDAR. **(B,C)** Rotated views of the predicted EAR-20 structure highlighting key residues involved in its interaction with the receptor. Contacts include Asp524 on the GluN2B subunit, and Ser189 and Ser773 on the GluN1 subunit. **(D)** Quantification of EAR-20 (100 µM) potentiation on GluN1-GluN2B wild-type receptors and various single-, double-o triple-mutants mutants (S773G, D524G, S189G) showing that removal of GluN1-Ser773 significantly reduces EAR-20-induced potentiation in all cases. Data represent mean ± SEM with individual point denoted as open circles. Data is shown as mean ± SEM; individual recordings are represented as open circles. Statistical analysis was performed using Kruskal–Wallis test followed by Dunn’s *post hoc* tests. Significance is as follows: p < 0.05 (*), p < 0.01 (**), and “ns” is not significant.

First, given that the secondary structure of EAR-20 has not been determined, we employed three different computational tools to predict its most probable three-dimensional conformation. With each structural prediction tool, we obtained scoring values to estimate the quality of the generated models (I-TASSER reported C-scores, Modeller used DOPE energy scores, and PEP-FOLD applied the sOPEP energy function). Among the predicted conformations with these complementary methods, the model generated by PEP-FOLD4 of EAR-20 structure was therefore chosen for docking based on its favorable sOPEP energy score and Ramachandran plot validation. This structure was therefore selected for subsequent molecular docking experiments (Figure 8A). Docking simulations predicted that EAR-20 binds at the interface between the GluN1 and GluN2B subunits within the LBD, as evident by the estimated lower binding energy required of −7.3 kcal/mol and a great number of hydrogen bonds and hydrophobic interactions (Figures 8A,B). Interestingly, several studies have described this region to be crucial for PAM activity (Esmenjaud et al., 2019).

These *in silico* results revealed multiple contacts between EAR-20 and receptor residues, including Ser773, Ser189, and Asn161 from GluN1, and Asp524 and Ser520 from GluN2B (Figure 8B, right; Figure 8C). Among these, serine 773 (S773) of GluN1 subunit was centrally located within the binding interface, suggesting a primary role in EAR-20 interaction. Moreover, according to this model GluN1-S773 might be interacting with two EAR-20 residues, Glutamine 9 (Gln 9) and Glutamate 7 (Glu 7) by means of 4 different hydrogen bonds.

Based on the docking results, GluN1-Ser773 residue was prioritized for site-directed mutagenesis. We changed the serine to a glycine (S773G) to subsequently perform electrophysiological assays and test the potential binding role of this GluN1 residue in the interaction with the EAR-20 peptide. Substitution of Ser773 with glycine led to a significant reduction in EAR-20 potentiation (from 136.8% ± 19.6% to 48.2% ± 10.4%; Figure 8D), confirming its role in EAR-20 interaction.

To assess whether Asp524 of GluN2B, which exhibited the second highest number of predicted interactions with EAR-20 (two contacts), plays a crucial role in the peptide’s modulatory effect, we generated a double mutant combining GluN1-S773G and GluN2B-D524G. The potentiation in this double mutant remained at 52.2% ± 8.4%, comparable to the effect observed with the single GluN1-S773G mutation, showing that the loss of interaction with Asp524 does not further reduce EAR-20 activity, and highlighting Ser773 as a more critical residue. To further confirm that Asp524 is less important than Ser773, we mutated another residue of GluN1, Ser189, which was predicted to have only a single contact with EAR-20, and combined it with the Asp524 mutation. In this double mutant (GluN1-S189G + GluN2B-D524G), the potentiation remained similar to that observed in the wild-type receptor (158.2% ± 8.8%), indicating that disruption of Asp524 alone does not significantly impair EAR-20 activity in the absence of Ser773 modification.

Finally, a triple mutant was constructed by introducing the GluN1-Ser773Gly mutation into the GluN1-Ser189Gly + GluN2B-Asp524Gly background. In this case, potentiation was again reduced in a similar extent as for GluN1-S773G or GluN1-S733G+GluN2B-D524G, confirming that one of the critical determinants for EAR-20’s positive allosteric modulation is the interaction with GluN1-Ser773.

### Modulatory effects of EAR-20 on NMDARs carrying loss-of-function mutations

Due to the observed activity of EAR-20 as a positive allosteric modulator on wild type NMDARs, we decided to explore its potential to enhance and thus rescue NMDAR hypofunction by testing its effect on two representative pathogenic variants associated with GRIN disorders.

**FIGURE 9:**
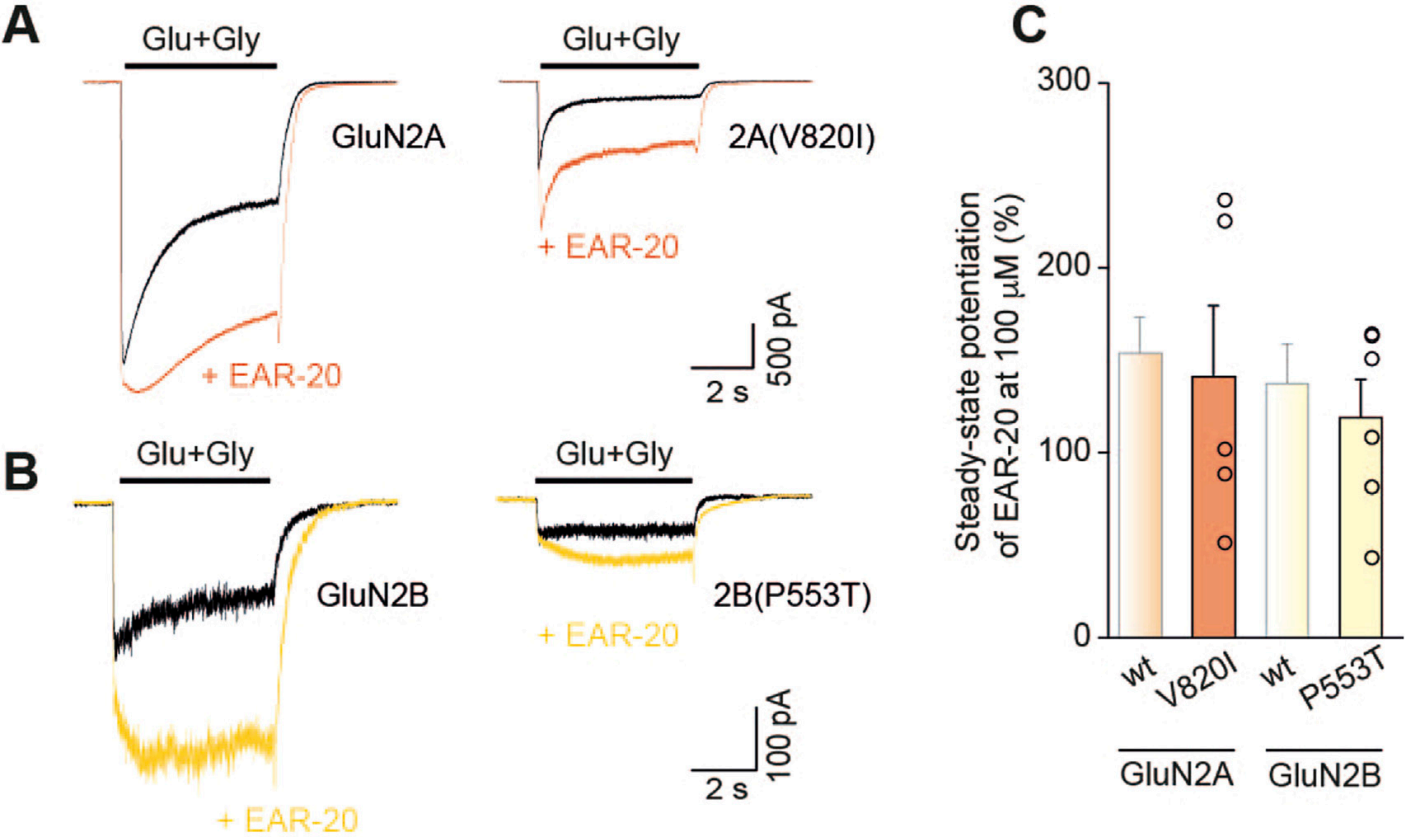
EAR-20 enhances NMDAR function in receptors harboring loss-of-function mutations. **(A)** Representative whole-cell recordings from HEK-293T cells expressing wild-type GluN1/GluN2A or mutant GluN1/GluN2A-V820I receptors, showing currents evoked by rapid application of 1 mM glutamate and 50 µM glycine, in the absence (black traces) or presence of 100 µM EAR-20 (orange traces). **(B)** Same as in A, but for wild-type GluN1/GluN2B and mutant GluN1/GluN2B-P553T receptors (yellow traces). **(C)** Quantification of EAR-20-induced potentiation at steady-state (100 µM) for each receptor type. Bars show mean ± SEM; individual values are indicated as open circles. No statistical significance was found by Mann Whitney U-test for both comparisons GluN2Awt vs. V820I and GluN2Bwt vs. P553T (p > 0.05).

We first examined the loss-of-function (LoF) GluN2A-V820I variant, previously shown to markedly reduce current amplitudes and to accelerate desensitization, consistent with hypofunctional receptor phenotype (Miguez Cabello, 2020; García-Recio et al., 2021; García-Recio et al., 2021), changes that were replicated in this study (Figure 9A; black traces). Upon co-application of EAR-20, NMDAR-mediated currents in the mutant receptor were significantly increased (Figure 9A, right trace in orange) in a similar manner as the potentiation in wildtype receptors (146.6% ± 37.8% vs. 154.3% ± 19.0% for GluN2A-V820I and GluN2A-wildtype; p = 0.8795, Mann-Whitney U-test; Figure 9C). In addition to potentiating whole-cell current responses, the application of EAR-20 on the GluN2A-V820I variant also slowed receptor desensitization reducing its rate relative to agonist-only conditions.

As a prototypical LoF variant in the GluN2B subunit, we selected GluN2B-P553T, a mutation previously reported to cause severe functional impairment, which has been partially rescued by other positive allosteric modulators in prior studies (Fedele et al., 2018; Soto et al., 2019). Notably, the percentage of potentiation induced by EAR-20 in this variant was comparable to that observed for wild-type GluN2B-containing di-heteromeric receptors throughout this study (119.3% ± 20.2% vs. 137.9% ± 21.0% for GluN2B-P553T and GluN2B-wildtype; p = 0.9044, Mann-Whitney U-test; Figure 9C).

These results provide proof of concept that EAR-20 or its derivatives could potentially be used in the future as compounds with potential therapeutic use, in particular with diseases caused by NMDAR hypofunctionality.

## Discussion

NMDARs play a critical role in excitatory neurotransmission in the CNS, being essential for the proper maturation and plasticity of neuronal synaptic contacts. Consequently, their dysfunction has been implicated in different neurological disorders (Capó et al., 2025). Accordingly, extensive research has long focused on identifying mechanisms to modulate NMDAR function in the context of pathologies involving either a loss- or gain-of-function. However, one of the main limitations has been the poor selectivity. The use of glutamate, its derivatives or glycine as strategies to enhance NMDAR function is not feasible due to their non-specificity over other receptors. Glutamate can also activate ionotropic and metabotropic glutamate receptors, while glycine is a primary agonist at inhibitory glycine receptors. A similar limitation applies to the use of antagonists or channel blockers, which often lack receptor and subunit specificity. For example, the NMDAR antagonist memantine that has been used in clinical assays (Lu and Nasrallah, 2018), and despite it is supposed to be selective for NMDARs, it cannot be really considered a highly selective drug, as it can also bind to other receptors and ion channels (Carrillo et al., 2025). Moreover, memantine does not completely discriminate between NMDAR subunits and its inhibitory potency varies depending on the subunit composition of the receptor (Kotermanski and Johnson, 2009).

However, the normal function of NMDARs can be enhanced by several molecules that act as PAMs, increasing the receptor’s responses to its natural agonists. Allosteric regulation is a crucial process that regulates the biological activity of proteins. In the case of membrane ionotropic receptors, allosteric modulation can alter ligand-binding properties, cause conformational changes, and affect activation, inactivation or kinetic behavior. In addition, PAMs offer several advantages as therapeutic agents since they do not usually exhibit off-target effects on unrelated receptors and even typically show greater selectivity towards one type of GluN2 subunit (GluN2A, GluN2B, GluN2C or GluN2D). This selective activity exhibited by PAMs arises, in most cases, from their ability to bind regions distinct from the ligand binding domain and the ion channel pore, both of which are highly conserved across NMDARs. As a result, PAMs are less likely to produce non-specific pharmacological effects, thereby reducing the risk of developing unwanted side effects (Burnell et al., 2019). This scenario has driven interest in identifying PAMS targeting NMDARs as potential therapeutic agents for addressing the cognitive deficits associated with NMDAR dysfunction.

In the present study, we have characterized the electrophysiological properties of EAR-20, a peptide based on a scrambled derivative of Conantokin-G–a peptide from cone snail venom that selectively targets NMDARs with promising therapeutic potential for neurological disorders (Jimenez, 2022). Initial trials with EAR-20 showed that unexpectedly enhanced NMDAR responses (Reyes-Guzman et al., 2017). Here, using whole-cell patch-clamp recordings in HEK293T cells expressing both diheteromeric GluN1-GluN2 and triheteromeric GluN1-GluN2A-GluN2B receptor assemblies, as well as hippocampal neurons, we characterize EAR-20 effects and demonstrate that it acts as a PAM of NMDARs by interacting with the ligand-binding domain of either GluN1 and GluN2B subunits. Its modulatory effect was dose-dependent and significantly enhanced both peak amplitude and steady-state NMDAR-mediated currents, displaying a clear subunit-dependent profile. The potentiating effect of EAR-20 likely stems from a reduction in receptor desensitization and stabilization of open-channel conformations.

As stated, the PAM effect observed for EAR-20 on steady-state NMDAR currents appears to be subunit-dependent. In particular, the peptide only partially potentiated GluN1-GluN2C NMDARs whereas it produced greater effect in receptors containing GluN2A, -B, or -D subunits, which showed a potentiation ranging from 100% to 150%. Molecular docking analysis using the GluN1/GluN2B receptor structure suggested that the leucine residue of the EAR-20 interacts with the Asp524 of the GluN2B subunit, which is conserved in all GluN2 subunits. Interestingly, the two amino acids immediately upstream of Asp524 vary slightly among subunits. GluN2A and GluN2B, which were the subunits showing the strongest EAR-20 potentiation, each contain two valines at these positions. In contrast, GluN2D contains a valine and an isoleucine, while GluN2C has two isoleucines and shows the weakest potentiation (∼25%). The presence of bulkier isoleucine residues may introduce steric limitations, thereby reducing EAR-20 binding affinity or altering binding conformation, ultimately diminishing its allosteric effect. These findings suggest that local side-chain composition near Asp524 may critically influence the efficacy of EAR-20.

The aforementioned residue Asp524 from GluN2B, and all other residues identified by molecular docking interacting with EAR-20, are located within the LBD of either the GluN2B or GluN1 subunit. These findings align with recent literature reports, suggesting that PAM activity is not directly related to the amino-terminal domains ATDs as previously thought, but may instead result from compound interactions across the whole receptor structure (Geoffroy et al., 2022). In this context, GluN1-GluN2 LBD interface has been proposed as a key site for allosteric modulation, potentially contributing to the subunit-selective activity of PAMs (Khatri et al., 2014; Hackos et al., 2016; Volgraf et al., 2016; Bledsoe et al., 2019).

Among the other residues predicted to interact with EAR-20, Ser773 of GluN1 exhibited the highest number of contacts, forming four distinct interactions within the binding pocket. This residue is centrally located in the receptor-peptide interface and appears to anchor the peptide within the ligand-binding domain. Its functional importance was confirmed experimentally, as mutation of Ser773 alone was sufficient to reduce potentiation by EAR-20 by approximately two-thirds. In contrast, Asp524 of GluN2B, which formed two predicted interactions, contributed less critically to the modulation, as indicated by the lack of further reduction in potentiation in the double mutant. Additional residues such as Ser189 and Asn161 of GluN1, despite their proximity, made fewer contacts and appear to play only minor or supportive roles in peptide binding. Despite the central role of Ser773, site-directed mutagenesis did not fully eliminate the potentiating activity of EAR-20. Approximately one-third of the original effect remained even after mutating the residues predicted to participate in peptide binding with higher number of interactions. This residual activity may reflect contributions from additional, less prominent residues beyond Ser773 or Asp524, whose role was not emphasized by the docking predictions but may nonetheless be functionally significant. Essentially, our objective was not to exhaustively map every residue critical for peptide action, but to localize the binding site within the receptor’s overall topology. Having now experimentally validated the GluN1–GluN2B ligand-binding domain interface as the primary interaction site for EAR-20, further studies will be required to refine the specific molecular determinants of this interaction. Thus, the persistent modulation observed may result from unmutated residues that act in concert with Ser773, or alternatively, from the peptide binding to additional allosteric sites within the receptor complex.

An interesting and consistent observation throughout this study in recordings using EAR-20 was the presence of a transient rise in current following washout of the extracellular solution containing both agonists and the EAR-20 (Figures 2, 3, 5, 7, 9). This tail-like response, marked by a brief increase in NMDAR-mediated current only upon removal of the modulatory EAR-20, has also been described for other positive allosteric modulators such as pregnenolone sulfate (Chopra et al., 2015). Similar tail-like current responses upon compound washout have also been reported for other modulators that are not classified as PAMs. For example, it has been described a transient inward current following the removal of 9-aminoacridine (9-AA), a channel blocker, in GluN1-GluN2A receptors (Wang et al., 2021). Although the underlying mechanism remains unclear, several hypotheses may explain this effect. One possibility is that the peptide remains transiently bound to the receptor for a short time after the agonist’s removal, briefly stabilizing a conformation that favors channel opening. Alternatively, EAR-20 may induce short-lived structural changes that persist momentarily after its removal, leading to a delayed and brief reactivation of the receptor. In any case, tail currents seem to reflect transitional receptor states during recovery from modulator binding, rather than residual agonist activity. Interestingly, the existence of similar tail-like responses from mechanistically distinct agents–such as PAMs and channel blockers–may represent an example of functional degeneracy (Edelman and Gally, 2001), a phenomenon in complex systems whereby different mechanisms converge on the same output. This raises the possibility that tail responses reflect a common emergent property of NMDAR modulation, independent of the precise molecular trigger. Regardless of the exact mechanism, this washout-induced potentiation further supports the allosteric nature of EAR-20 and aligns with observations made for other NMDAR positive modulators with similar pharmacological profiles (Chopra et al., 2015; Chopra et al., 2017).

Given that EAR-20 enhances macroscopic currents and reduces receptor desensitization, we have studied whether these effects are related to changes in single-channel properties, such as open probability, mean open time, burst duration or mean amplitude since single-channel recordings provide the resolution necessary to dissect these parameters and gain mechanistic insight into how EAR-20 modulates receptor gating at the molecular level (Parsons et al., 1993). In our experiments, EAR-20 markedly reduced the time NMDARs spent in the long-closed conformations (states previously linked to receptor desensitization) suggesting that the peptide reduces receptor entry into these desensitized states. Concurrently, the duration of open state 2 was significantly prolonged, reflecting a stabilization of longer channel openings. These changes in gating kinetics are consistent with a shift in the conformational equilibrium toward more active receptor states, reinforcing the hypothesis that EAR-20 acts by favoring gating modes with higher activity. These results support the idea that EAR-20 enhances receptor activity not only by promoting channel opening, but also by limiting desensitization, a dual mechanism likely contributing to its robust potentiation observed.

Our single-cannel analysis identifies four distinct closed states, although the presence of five closed states has commonly been reported in studies fitting native receptors to five exponential components (Banke and Traynelis, 2003; Chopra et al., 2017; Amin et al., 2023). Nevertheless, other studies have also reported good fits using four (Erreger and Traynelis, 2008) or even three exponential components (Esmenjaud et al., 2019). The C5 (closed time 5) is the longest shut state, typically lasting between 1 and 2 s, and represents a very small fraction of the overall distribution (approximately 0.1% when fitting to five exponential components), making it particularly difficult to detect. Our observation of only four clear closed states rather than the canonical five could be attributed to the presence of multiple channels within the recording patch. In this context, detecting the long-closed states as C5 can be challenging due to overlapping openings, which interrupt those long-closed times and lead to underestimation. In multi-channel recordings, a channel may reopen before a prolonged closure is fully observed, or another channel may open, further masking C5 events. Even in supposedly unique single-channel recordings of NMDARs, some closed states might not be observed as reported for the gain-of-function variant GluN2A(P552R), where desensitized conformations are disfavored, and the fifth closed state component is not observed (Iacobucci et al., 2022). Likewise, the enhanced channel open probability induced by EAR-20 could reduce the occurrence of long shut periods, contributing to the non-detection of C5. Additionally, the limited number of events (∼50,000) in our dataset may constrain the ability to resolve such low-probability states. However, despite these caveats, the kinetic effects of EAR-20 were clearly evident, as the peptide shifted the closed dwell-time distribution, reducing the time spent in closed states. Notably, the most affected shut components corresponded to the closed states associated with macroscopic desensitization of the receptor, suggesting that as the mechanism by which EAR-20 enhances receptor activity.

In this work, we also extended our findings from recombinant expression systems to a native neuronal context to evaluate the potential physiological relevance of EAR-20 modulation of NMDARs. Our data clearly show that the potentiation observed in recombinant receptors can be extrapolated to neuronal receptors, indicating that EAR-20 is capable of enhancing currents mediated by neuronal populations of NMDARs. However, when agonists were co-applied with EAR-20 in cultured hippocampal neurons, the observed potentiation was lower compared to that seen in GluN1-GluN2A and GluN1-GluN2B di-heteromeric receptors. This reduced effect may be explained by the predominant presence of GluN1-GluN2A-GluN2B tri-heteromeric NMDAR in the hippocampus of neonatal and adult mice (Rauner and Köhr, 2011), which, as observed in our recombinant system experiments, display significantly lower sensitivity to EAR-20. Supporting this, previous studies have demonstrated that tri-heteromers differ substantially from di-heteromers in key pharmacological properties, including glutamate desensitization and responses to allosteric modulators (Hansen et al., 2014; Stroebel et al., 2018; Gibb, 2022). For example, similar to what it is observed with EAR-20, spermine strongly potentiates GluN2B-containing di-heteromers but fails to enhance triheteromeric NMDARs composed of GluN1, GluN2A, and GluN2B subunits (Kellner and Berlin, 2024). Also, memantine appears to have a lower affinity for triheteromeric NMDARs containing both GluN2A and GluN2B subunits compared to those containing only GluN2B subunits (Chen et al., 2020). The peak and steady-state potentiation levels recorded in hippocampal neurons (54% and 57%, respectively) were substantially lower than those observed for di-heteromers, yet higher than those measured in GluN2A/2B tri-heteromeric receptors. These results suggest the presence of a mixed population of NMDARs in native neurons, with tri-heteromers representing the major component and a minor contribution from GluN2B-containing di-heteromers. Such receptor composition could account for the intermediate pharmacological profile of EAR-20 observed in hippocampal neurons. This reinforces the notion that triheteromeric assemblies exhibit distinct pharmacological profiles and may underlie the reduced potentiation observed with EAR-20 in hippocampal neurons.

Another notable difference with the recombinant receptor experiments was evident when analyzing spontaneous activity of hippocampal neuronal cultures: the inactivation kinetics of the EPSCs did not appear to be affected by EAR-20, in contrast to the observed in expression systems where deactivation was clearly slowed. This difference can be easily attributed to the fact that, under physiological conditions, the glutamate transient in the synaptic cleft is brief–in the order of a few milliseconds–so that receptors undergo deactivation without entering a desensitized state (Clements et al., 1992; Clements, 1996). Therefore, in EPSCs, the potentiating effect of EAR-20 would be primarily evident on the peak current, while the modulation of steady-state currents, as observed under continuous agonist application, would not be detectable. The observed 43% potentiation of the EPSC peak is consistent with the peak enhancement seen in triheteromeric receptors expressed in HEK cells, further supporting the idea that EAR-20 is acting on this receptor population present at the synapses in neurons. Importantly, the significant potentiation observed in NMDAR-mediated EPSCs in cultured hippocampal neurons, in conditions where only EAR-20 was added to the bath, strongly suggests that EAR-20 is capable of modulating receptors activated by endogenous levels of agonists during synaptic transmission. Furthermore, the increase in EPSC amplitude in the absence of changes in event frequency supports the interpretation that this effect is primarily postsynaptic, consistent with a direct modulation of receptor function rather than a presynaptic change in release probability.

An interesting aspect worth highlighting is that, the initial goal of this study (as shown in Figure 1) was to evaluate whether EAR-20 peptide alone was sufficient to activate NMDARs based on previous observations by Reyes-Montaño and colleagues, who reported that the solely application of EAR-20 onto hippocampal neurons elicited NMDAR-mediated currents (Reyes-Guzman et al., 2017)]. In that work, hippocampal neurons exposed to the EAR-20 (SEQ ID No: 4) displayed inward currents consistent with NMDAR activation, even in the absence of added glutamate or glycine. These early findings suggested a potential partial agonistic or modulatory role for EAR-20 at the NMDAR complex. Now, in this work, and beyond the clear PAM activity, we have found that EAR-20 peptide appears to partially activate NMDARs even in the virtual absence of natural co-agonists (glutamate and glycine). We are not aware to any compound capable of activating NMDARs in the absence of both agonists. The currents observed upon EAR-20 only application could be due to a favored conformational state elicited by EAR-20 binding, in which a certain degree of receptor gating becomes possible even in the absence of glutamate and glycine binding. However, it is also highly likely that contaminating traces of glycine might be responsible for the effect of EAR-20, since in our experiments, the addition of glycine potentiated EAR-20-induced agonistic response. This possibility is supported by the fact that, based on our experiments, the application of glutamate alone did elicit NMDAR-mediated responses, which could similarly be attributed to residual contaminating glycine in the recording solution. This observation is not unprecedented as glutamate alone has been previously shown to weakly activate NMDARs, although far less efficiently than with the simultaneous binding of glutamate and glycine (Johnson and Ascher, 1987). Additionally, fluid shear stress has been reported to activate NMDARs through mechanical deformation of the receptor or its membrane environment, independent of glutamate binding (Maneshi et al., 2017), which supports the fact that EAR-20 activates the receptor alone. However, in Maneshi *et al.*, the absence of glycine is not explicitly confirmed. Therefore, the conformational change enabling channel opening in that context may resemble the one seen for EAR-20, where glycine could facilitate gating and ion permeation through the channel in the absence of glutamate. Thus, although there is evidence that point that EAR-20 may exert agonist-like effects on its own, the requirement of glycine cannot be ruled out.

Since proper NMDAR activity is essential for CNS function, understanding and manipulating allosteric regulation is of critical importance for the development of new therapies targeting NMDAR dysregulation. Positive allosteric modulators of NMDARs have been shown to successfully address NMDAR hypofunction (Tang et al., 2020; Tang et al., 2023; Santos-Gómez et al., 2025) as well as exert cognitive-enhancing and antidepressant effects in animal models (Collingridge et al., 2013), thus making them promising targets for drug development. In this study, we demonstrate that EAR-20 enhances the activity of NMDARs harboring pathological mutations associated with receptor loss of function. Through its specific positive modulation of NMDARs, EAR-20 may serve as a starting point for the design and optimization of more potent and selective modulators. Although systemic administration of EAR-20 – a 17-amino-acid peptide–may be limited by its inability to cross the blood-brain barrier, alternative delivery strategies like intrathecal injection may offer a feasible route. Nevertheless, these results provide a valuable proof of concept. Compounds targeting the EAR-20 binding site or smaller molecules interacting with the serine 773 of GluN1 could be developed in the future as therapeutic agents for disorders caused by NMDAR hypofunction. These findings contribute to the greater effort to develop novel treatments for neurological conditions involving NMDAR hypofunction, which underlies a wide range of neuropsychiatric and neurodevelopmental disorders, including schizophrenia, autism, epilepsy, ADHD, depression or Alzheimer’s disease (Escamilla et al., 2024; Tumdam et al., 2024; Forouzanfar et al., 2025).

## Data Availability

The raw data supporting the conclusions of this article will be made available by the authors, without undue reservation.
